# Deep time perspective on turtle neck evolution: chasing the *Hox* code by vertebral morphology

**DOI:** 10.1038/s41598-017-09133-0

**Published:** 2017-08-21

**Authors:** Christine Böhmer, Ingmar Werneburg

**Affiliations:** 1 UMR 7179 CNRS/MNHN, Muséum National d’Histoire Naturelle, 57 rue Cuvier CP-55, 75005 Paris, France; 2 Senckenberg Center for Human Evolution and Palaeoenvironment at Eberhard Karls Universität, Sigwartstr, 10, 72076 Tübingen, Germany; 3 Fachbereich Geowissenschaften, Eberhard Karls Universität, Hölderlinstraße 12, D-72074 Tübingen, Germany; 4 Museum für Naturkunde, Leibniz-Institut für Evolutions- und Biodiversitätsforschung an der Humboldt-Universität zu Berlin, Invalidenstraße 43, 10115 Berlin, Germany

## Abstract

The unparalleled ability of turtle neck retraction is possible in three different modes, which characterize stem turtles, living side-necked (Pleurodira), and hidden-necked (Cryptodira) turtles, respectively. Despite the conservatism in vertebral count among turtles, there is significant functional and morphological regionalization in the cervical vertebral column. Since *Hox* genes play a fundamental role in determining the differentiation in vertebra morphology and based on our reconstruction of evolutionary genetics in deep time, we hypothesize genetic differences among the turtle groups and between turtles and other land vertebrates. We correlated anterior *Hox* gene expression and the quantifiable shape of the vertebrae to investigate the morphological modularity in the neck across living and extinct turtles. This permitted the reconstruction of the hypothetical ancestral *Hox* code pattern of the whole turtle clade. The scenario of the evolution of axial patterning in turtles indicates shifts in the spatial expression of *HoxA-5* in relation to the reduction of cervical ribs in modern turtles and of *HoxB-5* linked with a lower morphological differentiation between the anterior cervical vertebrae observed in cryptodirans. By comparison with the mammalian pattern, we illustrate how the fixed count of eight cervical vertebrae in turtles resulted from the emergence of the unique turtle shell.

## Introduction

The neck is a pivotal feature of tetrapods due to its involvement in a number of vital functions, such as feeding and locomotion as well as sexual display and combat behavior (“necking”)^1^. Furthermore, in turtles (Testudinata), the cervical vertebral column is involved in a defensive mechanism^2^. Their head-neck-system is unique since it can be protected against predators through the unparalleled ability to retract the head and neck inside the shell. Also reconstructed for extinct forms, neck retraction is possible in three different modes: by a simple ventrolateral bend as seen in stem turtles such as the Late Triassic stem turtle *Proganochelys quenstedti* ^3^, by horizontal neck retraction in living Pleurodira, and by vertical neck retraction in living Cryptodira, with the modes in extant turtles being characterized by a complex double-bend^4, 5^. The distinct neck retraction mechanisms are associated with specializations in vertebral morphology facilitating respective mobility in the cervical vertebral column^6–9^. Whereas the cervical vertebrae of stem turtles are only relatively little specialized in morphology, the side-necked motion in pleurodiran turtles is achieved by a highly specialized mid-cervical region and the hidden-necked motion in cryptodiran turtles is characterized by a highly specialized posterior cervical region^5, 6, 9, 10^. The mobility of the turtle neck might have had a crucial influence on shaping the temporal skull region in turtles^2^.

The formation of the axial skeleton is known to be mediated by *Hox* genes and evolutionary changes in the vertebral column have been associated with changes in the *Hox* gene expression patterns^11–13^. For instance, the expression of the *Hox-C6* gene governs the cervicodorsal transition in a variety of vertebrate species that differ in vertebral count, such as mouse, chicken, crocodile, turtle, and frog^11, 12, 14^. Even within the cervical vertebral column of amniotes, differences in the number of vertebrae and, thus, in the regionalization of the neck correspond to modifications in *Hox* gene expression domains (expansion of a *Hox* gene’s expression domain and/or a shift of gene expression)^15^. However, in contrast to other non-mammalian amniotes, the vertebral number is highly conserved in Testudinata^16^. Both stem and crown turtles have eight cervical vertebrae (CV). The only undoubted sister species of Testudinata, the carapace-lacking *Odontochelys semitestacea* has eight cervicals as well^17^. An extensive analysis of Late Triassic turtles recently provided evidence indicating that the “fixed” number of cervical vertebrae occurred after the formation of complete carapace^18^. Furthermore, the study identified a gradual change of the morphology of the eighth presacral vertebra from a dorsal to a cervical identity suggesting a homeotic transformation^18^. There is evidence that the presence of a carapace in turtles and other armored animals (e.g., placodonts, armadillos) is correlated with a decreased number of dorsal (trunk) vertebrae (DV) (ten DV in turtles)^16, 19, 20^. Derived turtle-specific traits in the expression of *Hox* genes have been correlated with a key specialization in their unique body plan, namely the carapace, which is based around the vertebrae and ribs of DV1 to DV10^14^. However, the conservative number of cervical vertebrae suggests a common *Hox* code in the neck for Testudinata in general, as it is hypothesized for mammals, which are highly constrained in cervical count (seven CV with only few exceptions)^21^. Although phylogenetically diverse, most mammals appear to display a common pattern of morphological differentiation within the neck and this is interpreted to reflect the common developmental regionalization^22–26^. It is not fully resolved yet (e.g., Varela-Lasheras *et al*.^27^), but the origin of the constraint in cervical count in mammals is regarded as non-adaptive, secondary consequences of developmental innovations tied to the adaptive respiratory complex (i.e., muscularized diaphragm; see discussion)^24^.

In contrast to mammals and despite the conservatism in vertebral count among turtles, there is significant functional and morphological regionalization in the cervical vertebral column in turtles according to the specific neck retraction modes. Since *Hox* genes play a fundamental role in determining the differentiation in function and form of vertebrae, we hypothesize genetic differences at least between Pleurodira and Cryptodira. Yet, we still lack complete information about the *Hox* gene expression pattern in the neck of turtles since only a partial *Hox* code for a cryptodiran turtle has been reported to date^14^. Therefore, our hypotheses could be tested with further genetic studies.

Reconstruction of *Hox* gene expression patterns based on vertebral morphology has become possible only recently^15, 28^. The correlation between anterior *Hox* gene expression and the quantifiable shape of the cervical vertebrae of living archosaurs (crocodile, alligator, and chicken) and mammals (mouse) has shown that changes in the expression of the underlying genetic code can be deduced solely from vertebral morphology^15, 25^. Furthermore, the correlation observed in extant crocodiles and birds permitted the reconstruction of the vertebral *Hox* code in an extinct relative that lacks preserved DNA and is known only from fossils remains^15^. Differences in the morphological subunits (modules) within the neck suggested that modifications in the expression of *Hox* genes have occurred during archosaur evolution^11, 15^.

The aim of the present study was to investigate the morphological modularity of the cervical vertebral column across living and extinct turtles in order to address the following questions: (1) is there a common modular pattern in the neck of stem turtles representing the ancestral configuration for turtles? (2) do Pleurodira and Cryptodira differ in morphological modularity of the cervical vertebral column; and as a consequence thereof (3) does the modular pattern in the neck of Pleurodira and Cryptodira represent their respective neck retraction modes? (4) does the reconstructed *Hox* code for turtles reflect their unique body plan?, and if so (5) can the associated developmental innovations of this lineage help explaining the constraint in vertebral count in turtles? The analyses provide new insights into the *Hox* code pattern of Testudinata as implied by vertebral morphological modularity. Ultimately, this improves our understanding of the evolutionary mechanisms responsible for the great morphological adaptability of the cervical vertebral column that has mediated the evolution of the unique turtle ‘body plan’^2^.

## Methods and Methods

### Taxa and phylogenetic relationships

**Figure 1:**
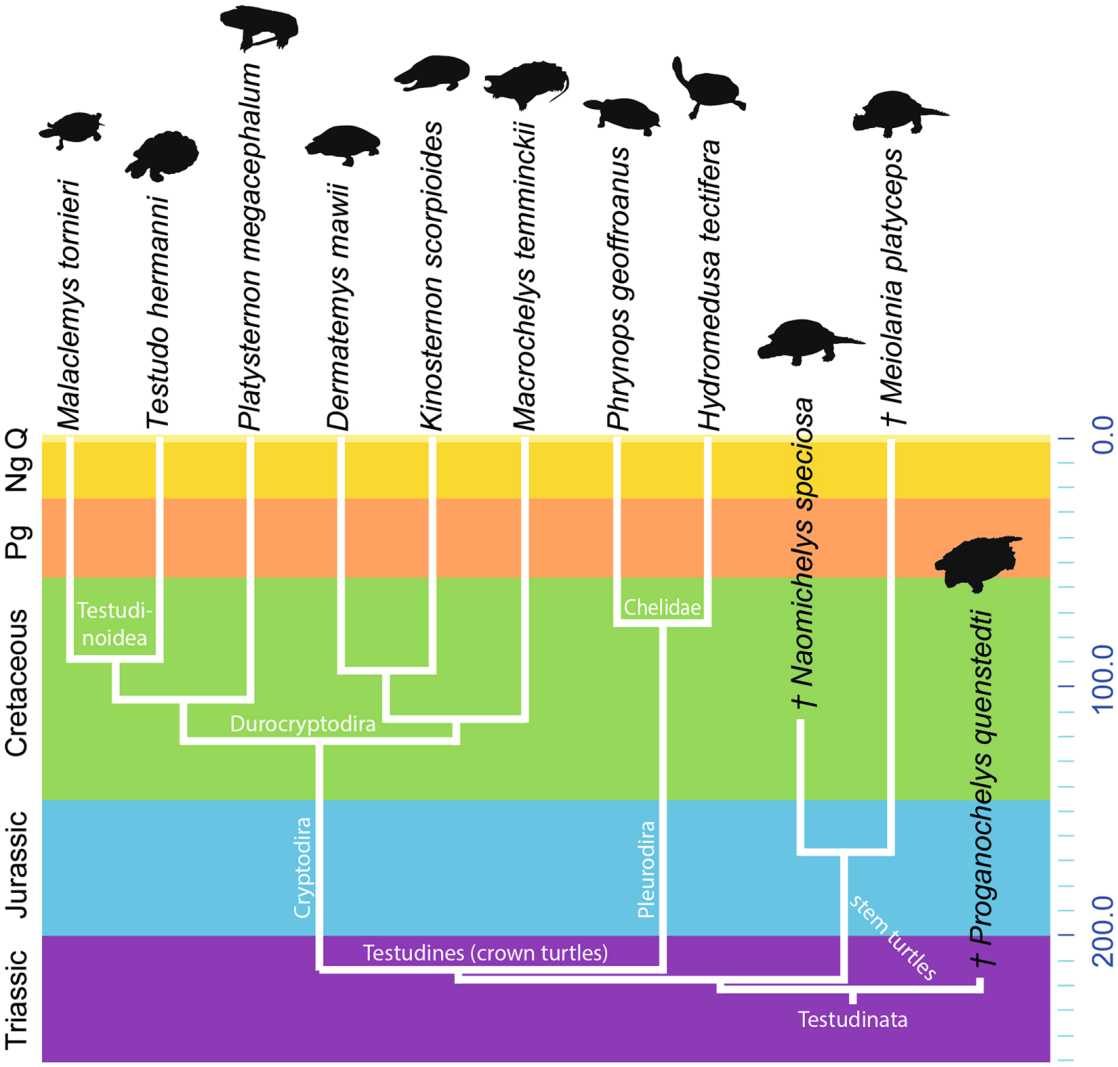
Taxon overview. Phylogenetic arrangement, divergence times, and stratigraphical distribution of the species analyzed in the present study. Divergence times follow Joyce *et al*. (2013).

The present study includes a total of 77 cervical vertebrae comprising three fossil turtles, all of them are stem members, and eight extant taxa (Supplementary Table S1). An overview of the species that have been morphologically analyzed and the phylogenetic framework is provided in Fig. 1. The analysis focuses on well-preserved forms (i.e., complete cervical vertebral column) that allow a comprehensive investigation.

### Morphometric analysis

The morphological variation within the cervical vertebral column of turtles was evaluated by a 3D geometric morphometric analysis and data gathered by Werneburg *et al﻿.* ^6^ following the procedure applied by Böhmer *et al*.^15^. To date, this procedure, as summarized below, represents the best possible method to identify morphological modules in vertebral series comprising less than 15 vertebrae as compared to the linear regression method described by Head & Polly^28^ since the general rule of thumb is a minimum number of at least 10 to 20 observations for a regression analysis^29^.

**Figure 2:**
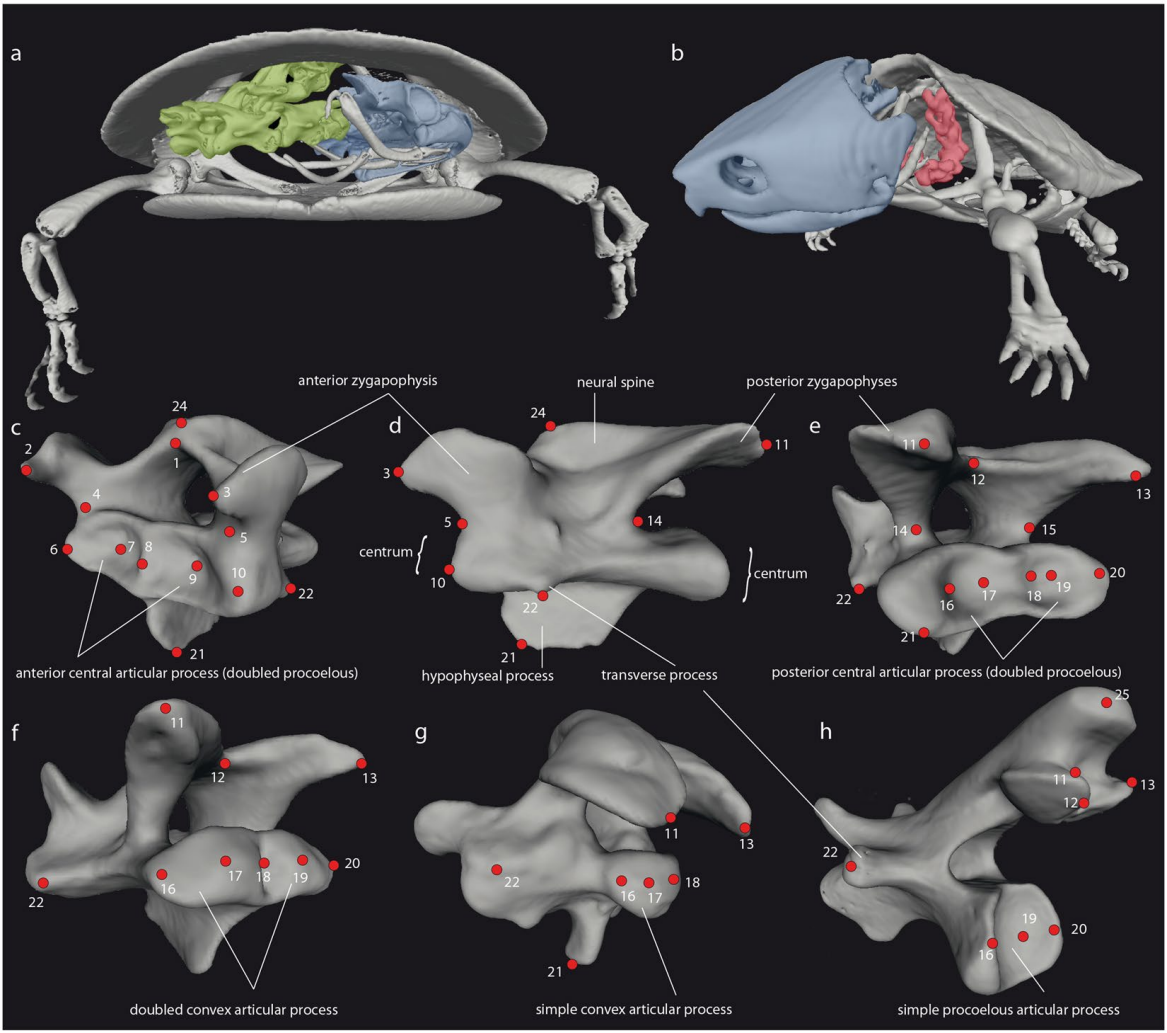
Vertebral morphology of turtles visualized using computer tomography. **(a**) Skeleton of the pleurodire turtle *Phrynops hilarii* in frontal view; the neck (green) is retracted. The skull (blue) fits below the anterior edge of the carapace. (**b**) The skeleton of the cryptodire turtle *Platysternon megacephalum* in frontolateral view. The neck (orange) is partly retracted. The skull (blue) is too large in this species to be fully retracted under the carapace. Cervical vertebra 7 of *P. megacephalum* is shown in frontolateral (**c**), left lateral (**d**), and posterolateral (**e**) view. This vertebra has double procoelous central articular processes. Vertebra 6 of this species has a doubled convex (**f**) and cervical 8 has a simple convex articular process (**g**) note the typical cryptodiran anatomy of the posterior zygapophyses in this last cervical vertebra). A simple procoelous articular process is shown for vertebra 7 of *Phrynops geoffroanus* (**h**). Numbers and red circles refer to the landmarks used in this study (Supplementary Table S2). For details on data sources see Werneburg *et al*. (2015a, b).

The morphological differences between the vertebrae within a cervical vertebral column were quantitatively analyzed via 3D landmark-based geometric morphometrics. The first cervical vertebra (atlas) was not included in the geometric morphometric analysis as it is highly modified and lacks recognizable serial homologies with postatlantal cervicals^8^, and thus, several landmarks cannot be applied to it. The 3D scans of the vertebrae (CV2 to CV8) were imported into the software Landmark^30^ and a total of 25 homologous landmarks were selected (Fig. 2, Supplementary Table S2).

Using the software PAST^31^, the 3D coordinates of each landmark set were superimposed using the General Procrustes Analysis (GPA). Next, a Relative Warps (RW) Analysis was performed to reduce the dimensionality of the dataset. With the applied settings this method is equivalent to a Principal Components Analysis (PCA) and reveals the similarity relationships among the cervical vertebrae for each taxon. The RW analysis constructs a morphospace in which shape variation can be quantified and the shape differences can be visualized with three-dimensional thin-plate splines. Via the cluster analysis using the single linkage algorithm in combination with the Euclidian similarity index the vertebrae were joined based on the smallest distance between them. Eventually, for the analyzed species, this resulted in the establishment of morphological subunit patterns (modules) of the cervical series.

### Genetic data and morphological proxies

**Figure 3:**
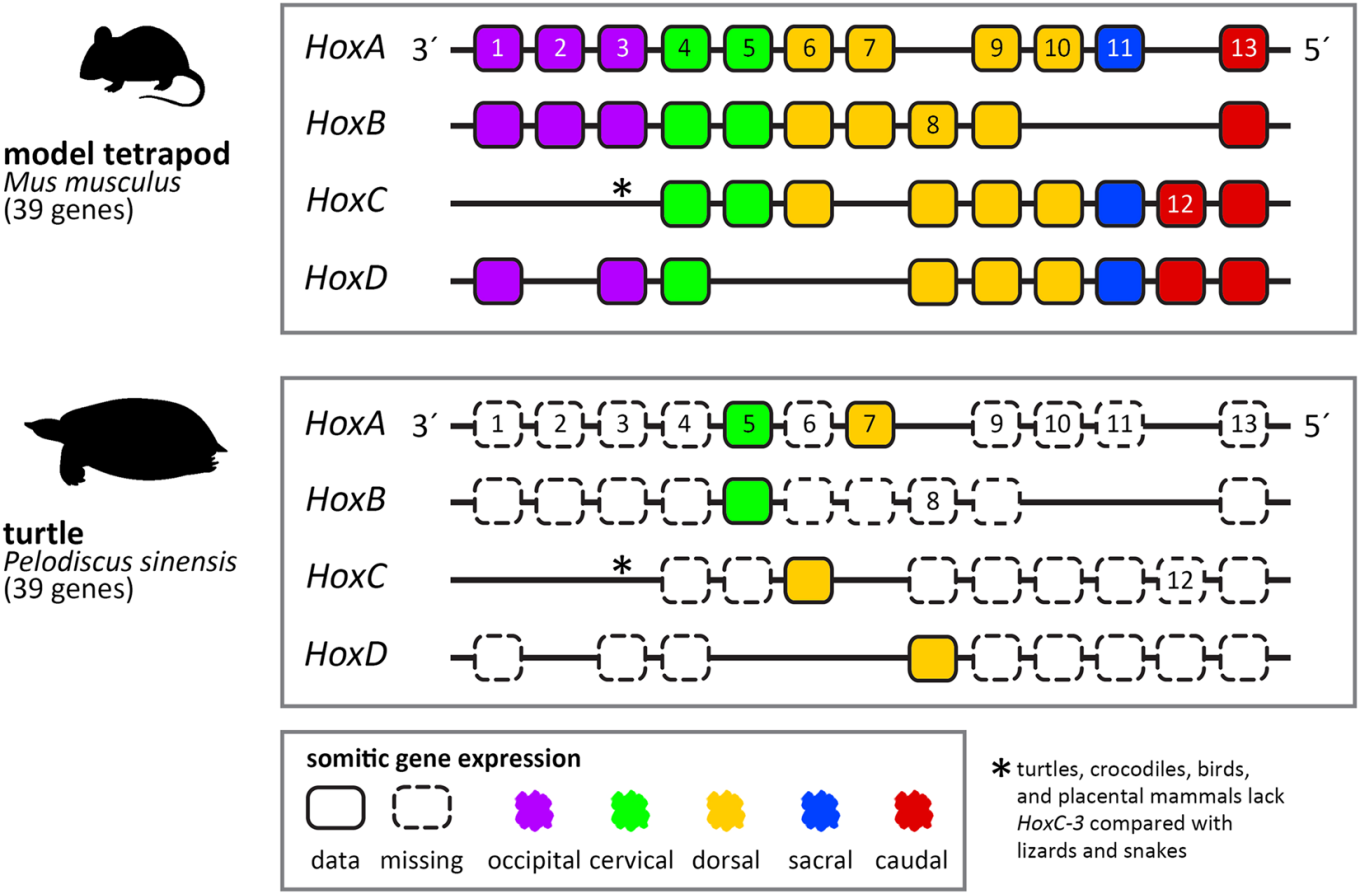
*Hox* gene inventory. Overview of the *Hox* gene inventory of the mouse *Mus musculus* and of the turtle *Pelodiscus sinensis* (information based on Liang *et al*. 2011) with indication of current knowledge of somitic *Hox* gene expression (information based on Ohya *et al*. 2005). The mouse is the most thoroughly studied animal concerning *Hox* gene expression and serves as model for tetrapods in general. The 39 *Hox* genes in tetrapods are arranged on four different chromosomes in four clusters (*HoxA*, *B*, *C*, *D*). The color coding indicates how gene groups map to the axial regions.

In contrast to lizards and snakes that retained the *HoxC-3* gene, turtles, crocodiles, birds, and placental mammals possess the same number of *Hox* genes arranged as 13 paralogue groups (PG) in four clusters (named A, B, C, and D)^32^. Previous studies reported on the *Hox* gene expression in the vertebral column of mice^12, 33^, chicken^12^, and crocodilians^11, 13, 15^. In turtles, the spatial expression of *Hox* genes is only partially known for the cryptodiran species *Pelodiscus sinensis* ^14^ (Fig. 3).

To formulate homology hypotheses^34, 35^ between *Hox* gene expressions in living archosaurs, the *Hox* gene expression patterns were compared in relation to vertebral morphology in crocodilians and birds^15^. The same *Hox* gene expression boundaries coincide with vertebral subunits^15^. Given the sister-taxon relationship of these two archosaur groups, this finding is most parsimoniously explained as implying homology between the modules^15^. These results from living archosaurs were then used as phylogenetic bracket^35^ to hypothesize *Hox* gene expression patterns from vertebral morphology in the most recent common ancestor of birds and crocodilians, and in a fossil representative of archosaurs^15^. On basis of the correlation between genomic control and phenotypic changes noted above, the present study of morphological variation of the cervical vertebrae served, in general, as a *Hox* gene expression pattern proxy.

## Results

**Figure 4:**
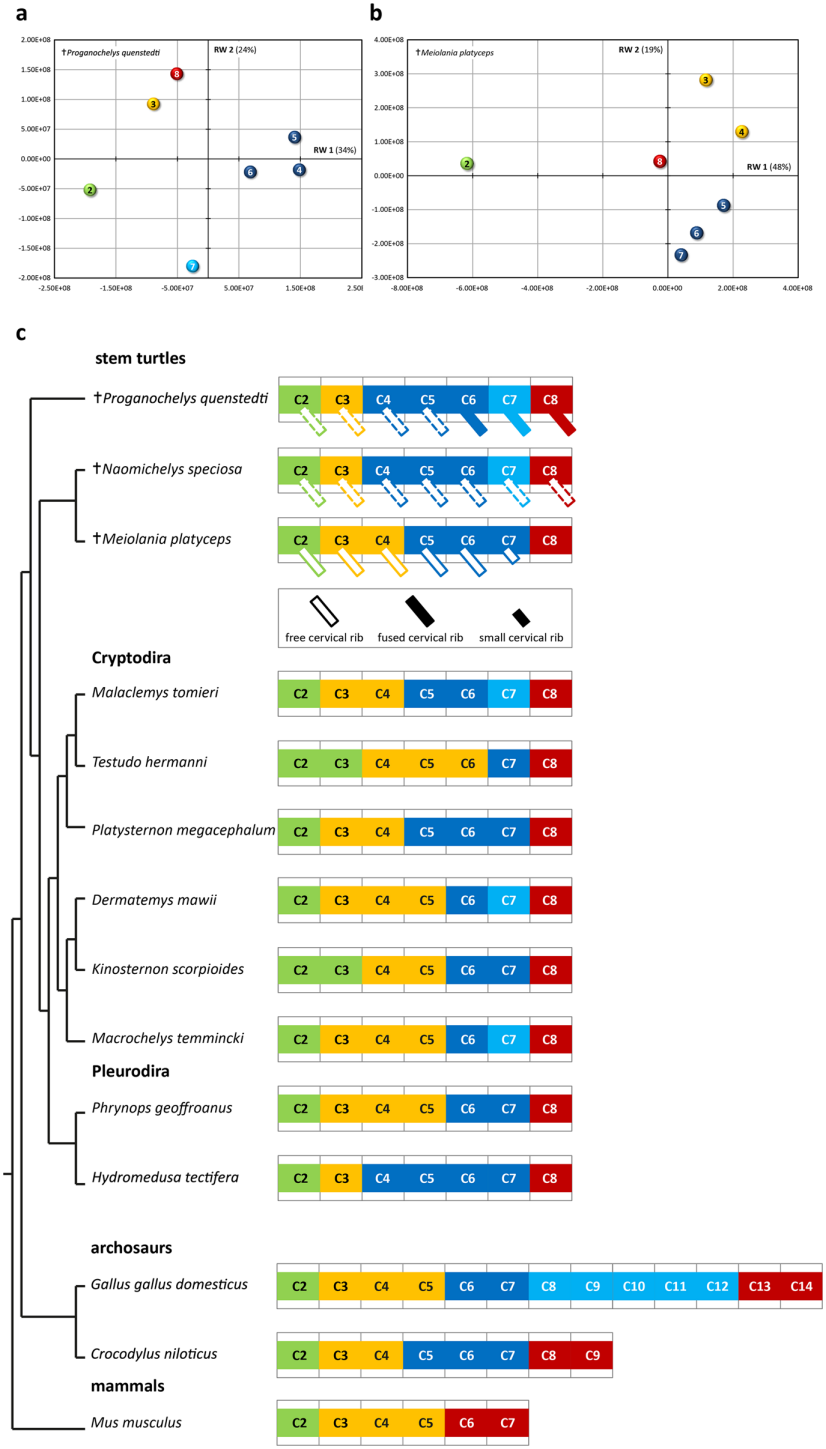
Morphological modularity in turtles. Result of the geometric morphometric analysis of (**a**) the Late Triassic turtle *Proganochelys quenstedti* and (**b**) the Pleistocene turtle *Meiolania platyceps* (Supplementary Fig. S1). Plots of the first two Relative Warp (RW) axes. (**c**) Morphological modularity of the neck in stem turtles, Pleurodira and Cryptodira in comparison to the morphological modularity of the neck in crocodile, chicken, and mouse (based on Böhmer *et al*. 2015b, Böhmer 2017). The color coding indicates morphological clusters of cervical vertebrae; i.e., vertebrae that are more similar to each other than to anterior or posterior vertebrae. In *Naomichelys*, cervical ribs are not preserved, but they are assumed because of the presence of diaphyses and parapophyses.

In all turtles analyzed, the morphometric analysis permitted discrimination of vertebrae in four or five different cervical regions. The common modular pattern comprises the axis (CV2, green in Fig. 4), an anterior (yellow), middle (dark blue), and posterior (red) region, but some species also display an additional midposterior (light blue) unit. The distribution of cervical vertebrae to the specific modules shows some variation among species.

### Morphological modularity of the neck in stem turtles

In the Pleistocene turtle *Meiolania platyceps*, the modular pattern includes the axis (CV2), two anterior (CV3 and CV4), three middle (CV5 to CV7) and one posterior (CV8) vertebra (Fig. 4). Whereas, in the neck of both the Late Triassic turtle *Proganochelys quenstedti* and the Early Cretaceous turtle *Naomichelys speciosa* (Fig. 1), the morphological units comprise the axis (CV2), one anterior cervical vertebra (CV3), three middle (CV4 to CV6), one midposterior (CV7), and one posterior (CV8) cervical vertebra.

### Morphological modularity of the neck in Pleurodira

Both pleurodiran turtles in the sample display a four-unit pattern in the cervical vertebral column (Fig. 4). In *Hydromedusa tectifera*, the middle region comprising CV4 to CV7 is the largest unit. In contrast, the anterior region including CV3 to CV5 is dominant in *Phrynops geoffroanus*.

### Morphological modularity of the neck in Cryptodira

The morphological analysis revealed four distinct cervical regions in the neck of three of the studied cryptodiran species (Fig. 4). The other three cryptodiran species have five different cervical regions in the neck (Fig. 4). The modular pattern shows some degree of variation. *Kinosternon scorpioides* and *Testudo hermanni* are unique among the whole turtle sample because the morphology of CV2 is not as distinct from CV3. Both vertebrae form one unit in these taxa.

## Discussion

Turtles display great morphological diversity within their cervical vertebral column^8^. Among others, this includes variation in the shape and relative height of the neural spines, the curvature and placement of the zygapophyses, and the character and degree of development of the transverse processes^6, 8^. Previous reports on the serial variation in the type of central articulation between the cervical vertebrae already documented a broad morphological regionalization of the turtle neck^8, 36–38^. However, early turtles, such as *Proganochelys quenstedti*, do not show signs of clear morphological regionalization since all cervical vertebrae have amphicoelous (biconcave) central articulations^39^. The present study is the first comprehensive investigation of morphological modularity in turtles, ranging from stem to modern taxa, considering the complete vertebral shape, which provides a foundation to study genetic mechanisms.

### *Hox* gene inventory in turtles

The involvement of the seven *Hox* genes of the paralogue groups (PG) 4 and 5 in mediating the formation of the cervical vertebral column of amniotes is well known reviewed by Mallo *et al*.^40^. And the eight *Hox* genes of the PG 6, 7, and 8 map to the dorsal (trunk) vertebral region marking the cervicodorsal transition^12^. In particular, *HoxC-6* is expressed at the first dorsal vertebra in amniotes with different cervical count^12^. Although the complete *Hox* gene inventory is known for *Pelodiscus sinensis* ^32^, we only know the spatial expression of few *Hox* genes yet (*HoxA-5, B-5*, *C-6*, *A-7,* and *C-8*)^14^. Nevertheless, we predict that the equivalent number of *Hox* genes from PG 4 to 8 is also expressed in the vertebral column of turtles. The conservation of *Hox* function across amniote species as shown by previous analyses^11, 12, 41^, the presence of the respective *Hox* genes in the genome of turtles^32^, and phylogenetic bracketing clearly support a model of functional equivalence (Fig. 3). A comparative survey identified only variation in the presence of the *HoxC-3* gene among amniotes^32^. Whereas squamates (represented by anole lizard, snake, gecko, and blind skink) possess the *HoxC-3* gene similar to osteoichthyian fishes^42, 43^ and amphibians, all other studied amniotes lack it^32^.

Misexpression experiments have elucidated the direct role of *Hox* genes in determining proper vertebral morphology^44, 45^. *Hox* gene expression is anteriorly distinct with mainly gradual posterior boundaries and negatively regulated by *Hox* genes posterior to them (posterior prevalence)^46^. *Hox* mutants usually show anomalies restricted to their anterior expression domain affecting the cervical vertebral or dorsal (trunk) vertebral phenotype. This allows us to narrow down where the *Hox* genes are most likely expressed in the neck of turtles. Targeted disruption of *Hox 4* and *Hox 5* genes results in transformations of the cervical vertebrae, and mutants of posterior *Hox* genes show anomalies in the anatomy of dorsal vertebrae^47–51^.

### Morphological modularity and vertebral *Hox* code in the neck

**Figure 5:**
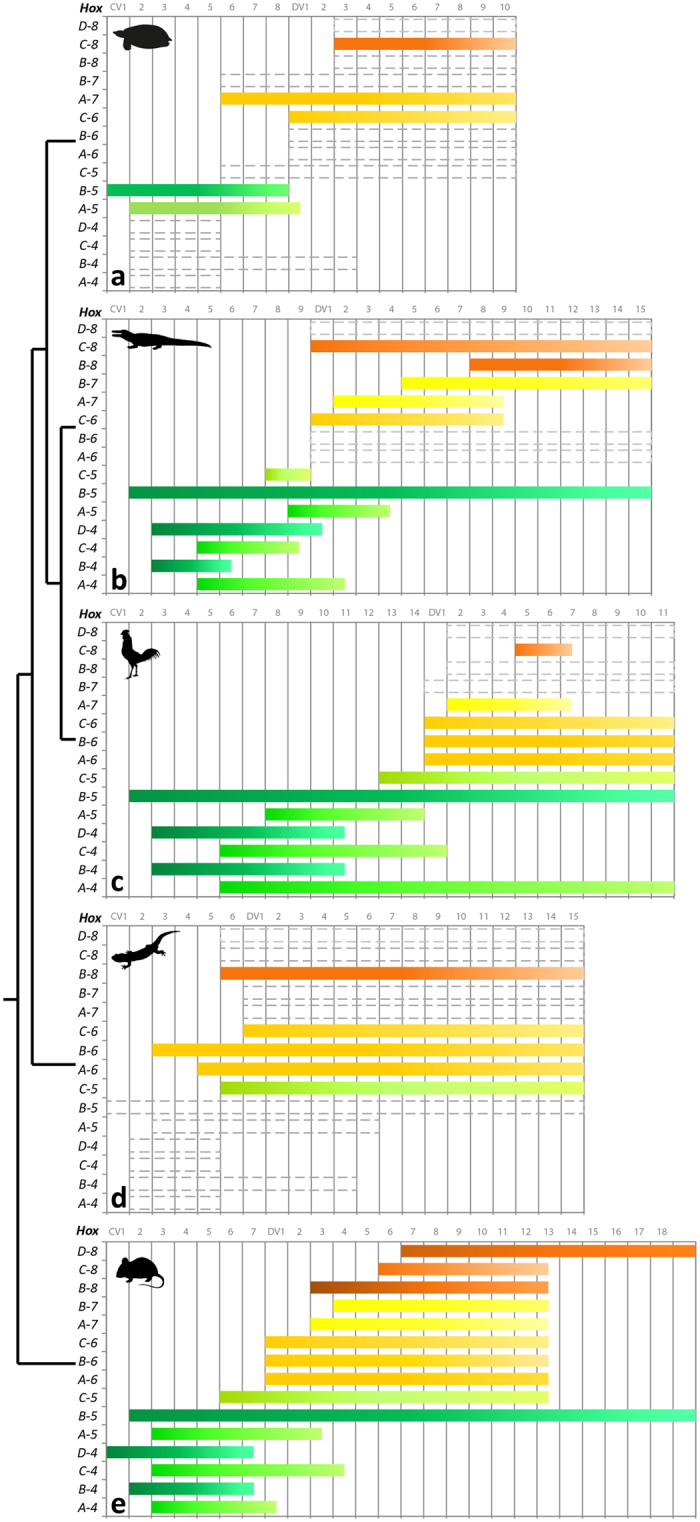
Summary of the somitic *Hox* code in amniotes. Modified from Böhmer *et al*. (2015a). Data for turtle (**a**) are based on Ohya *et al*. (2005) and for lizard (**d**) on Woltering *et al*. (2009). Information for crocodilians (**b**), chicken (**c**), and mouse (**e**) is based on Böhmer *et al*. (2015a). Anterior limits of expression are taken from determinations at relatively late stages. Posterior boundaries are not clearly defined.

As in lizards, crocodiles, chickens and mice, the first cervical vertebra of turtles develops from somites 5 and 6, the second cervical vertebra from somites 6 and 7^12, 14, 52, 53^. From anterior to posterior along the body axis of living archosaurs, the first *Hox* gene that is expressed in the cervical vertebral column is *HoxB-5* ^11, 13^ (Fig. 5b,c). Its expression starts at CV2 in crocodiles, chicken^11, 13^, and mouse^51^. There is no information available for the lizard. The anterior expression limit of *HoxB-5* correlates with the first morphological unit (axis) in the neck of archosaurs^15^. Although the present study detected a similar first unit comprising the axis, this morphological differentiation is not mediated by *HoxB-5* because, in turtles, the anterior expression limit of *HoxB-5* is shifted anteriorly by one vertebra^14^ (Fig. 5a). This indicates that - in contrast to archosaurs - the *HoxB-5* expression is also associated with the development of CV1 in turtles. And the genetic pattern may further suggest that the morphology of CV2 is not as distinct from the posterior cervical vertebrae as in other amniotes because they appear to share the same *Hox* code in the turtle. However, this interpretation is limited since we lack information on the expression pattern of the *Hox* genes of PG 4 (Fig. 5a). Yet, it is striking to note that the present analysis revealed a first morphological unit comprising CV2 and CV3 in at least two cryptodiran species, *Kinosternon scorpioides* and *Testudo hermanni* (Supplementary Fig. S1g,j). The morphology of these two cervical vertebrae is more similar to each other than to the posterior cervical vertebrae supporting the hypothesis that they share the same *Hox* code. The same observation has been made for *P. sinensis*. The atlas of this species diverges significantly in morphology from the post-atlantal vertebrae^54^, whereas the axis is similar in shape to the posterior cervical vertebrae^55^. The morphology of CV2 is always relatively different from CV3 in the studied pleurodiran species (Supplementary Fig. S1d,e). Therefore, the anterior *HoxB-5* expression in *P. sinensis* may be a cryptodiran-specific pattern. Further genetic analyses are required to provide direct evidence of the *Hox* code in a pleurodiran turtle (in which, as a side note, the atlas is not as derived in its morphology when compared to cryptodiran turtles).

Disruption experiments suggested that *HoxB-5* is involved in specifying the position of the limbs along the primary body axis because *HoxB-5* mutants revealed an anterior shift of the shoulder girdle^51^. It is striking to observe that the expression of *HoxB-5* is shifted anteriorly in turtles with their eight cervical vertebrae similar to the mouse, but in contrast to archosaurs with longer necks (crocodilians: nine CV, chicken: 14 CV) (Fig. 5a,b,c,e). The anterior expression limit of *HoxC-6*, however, is at the first dorsal vertebra (DV1) in all studied amniotes, including turtles, lizards, crocodiles, chickens, goose, and mice^11–14, 53^. In this regard, the developmental shift of the shoulder girdle in turtles is worth mentioning. It moves anterior and is laterally covered by the anterior trunk ribs later on^56^. Evolutionary this results in the unique position of the turtle shoulder within the body wall whereas it is situated outside in all other amniotes^57, 58^.

From anterior to posterior along the body axis of living archosaurs, the subsequent *Hox* genes that are expressed in the cervical vertebral column are of PG 4^11–13^ (Fig. 5b,c). The anterior expression limits of *HoxB-4* and *HoxD-4* are at CV3 in crocodiles and chicken^11–13^ and correlate with the second morphological unit (anterior) in the neck of these archosaurs^15^. The expressions of *HoxA-4* and *Hox-C-4* start at CV5 in crocodiles and at CV6 in chicken^11–13^ and correlate with the third morphological unit (middle) in the neck of archosaurs^15^. In the mouse, the expression pattern of the *Hox* 4 genes is different from living archosaurs^12, 59^ (Fig. 5e) indicating a possible mammal-specific pattern^11^. In lizard and turtle, there is no information available to date. The present morphological analysis revealed for all turtle species an anterior unit starting at CV3 (except for *Kinosternon* *scorpioides* and *Testudo hermanni*, as mentioned above) and a middle unit starting at CV4, CV5, or CV6 (Fig. 4). This may correspond to the expression of the *Hox 4* genes as described for archosaurs.

The last *Hox* genes that are expressed in the cervical vertebral column of living archosaurs are *HoxA-5* and *HoxC-5* ^11–13^ (Fig. 5b,c). *HoxC-5* starts at the penultimate cervical vertebra in Nile crocodile (CV8), chicken (CV13), and mouse (CV6)^11, 12^. It correlates with the last morphological unit (posterior module) in the neck of archosaurs^15^. In the lizard, the expression of *HoxC-5* is shifted posteriorly and starts at the last cervical vertebra (CV6)^53^. There is no genetic information available for the turtle. However, the present morphological analyses showed that all turtle species have a posterior morphological unit comprising solely the last cervical vertebra (CV8) (Fig. 4).

The spatial expression of *HoxA-5* starts at the last cervical vertebra (CV9) in crocodiles, but in the midposterior region of the cervical vertebral column at CV8 in the chicken^13^ (Fig. 5b,c). In the mouse, the anterior expression limit of *HoxA-5* is shifted anteriorly to CV3^13^. There is no information available for the lizard. In the turtle, the expression of *HoxA-5* starts in the anterior region of the cervical vertebral column at CV2^14^ (Fig. 5a). The anterior shift of *HoxA-5* expression in both mouse and turtle may be linked to the absence of free cervical ribs as *HoxA-5* is involved in mediating the development and suppression of cervical ribs^13–15, 48, 60^. Neck ribs where reduced in crown turtles, which is likely associated with the requirement to enable a greater mobility between vertebrae and to pack the neck into the body wall during neck retraction^3, 5–7, 9^.

Single mutation experiments in the mouse revealed that disruption of *HoxA-5* results in homeotic transformations of the axial skeleton confined between CV3 and DV2 and one of the most frequent morphological modifications is the emergence of a pair of ribs on CV7^45, 48, 60^.

In the bird, the expression of *HoxA-5* corresponds with an additional morphological unit (midposterior) detected in the cervical vertebral column and was interpreted to be a consequence of the long neck in the chicken (14 CV)^15^. The present morphological analysis revealed that some of the cryptodiran species have a midposterior morphological unit comprising only CV7, whereas none of the pleurodiran species shows an additional morphological unit (Fig. 4). This additional morphological unit, however, does not correlate with H*oxA-5* expression in turtles, but may be linked to the expression of another *Hox* gene. However, this hypothesis remains untested until further genetic studies will be performed. The presence of a separate vertebral unit in cryptodires appears to be related to the unique mode of neck retraction in this group. Compared to pleurodires, cryptodires show a vertical orientation of the retracted neck (Fig. 1b). To rotate the whole neck into the body, a highly modified anatomy of CV8 is necessary with highly elongated and curved posterior zygapophyses (Fig. 2g)^9^. In some cryptodires enormous movement is possible in the articulation of CV8 and DV1 enabling a total angular change of up to 180° ^3^. In order to enable even more angular change below the convex carapace and hence to further retract the neck into the shell, CV7 could show a particular variation in some taxa as the raw mobility between macerated vertebrae suggests^3^. Differences in the degree and mode of cryptodiran retraction may explain the great variation found in the modular pattern of their neck vertebrae, including the one additional module in two of them.

### Turtle-specific vertebral gene expression of *Hox* PG 6 to 8 and morphological modularity

The expression of the *Hox* genes of PG 6 is usually confined to the anterior dorsal vertebral column in amniotes (Fig. 5). The anterior expression limit of *HoxA-6*, *HoxB-6*, and *HoxC-6* is at DV1 in the mouse and in the chicken^12, 51, 53, 61, 62^. The expression of *HoxC-6* starts at DV1 in crocodiles as well^11, 13^, but there is no information available for the other two *Hox* 6 genes. In the lizard, the anterior expression limit of *HoxC-6* is at DV1, but the expression of *HoxA-6* and *HoxB-6* is more anterior and starts in the neck at CV5 (first rib-bearing cervical vertebra) and CV3, respectively^53^. The apparent link between *HoxA-6* and the formation of cervical ribs in the lizard is reinforced by the observation that mouse *HoxA-6* mutants acquire ribs at their cervical vertebrae^47^. In turtles, the anterior expression limit of only *HoxC-6* is known and it is at DV1 marking the cervicodorsal transition^14^. Considering the rib-promoting role of *HoxA-6* it is likely that its expression is confined to the dorsal vertebral column in turtles as seen in archosaurs. However, it has to be noted that cervical ribs develop in turtles as mesenchymal condensations and their chondrification is repressed later in development^7, 63^. Interestingly, in mouse *HoxB-6* mutants, the shoulder girdle is shifted anteriorly^51^, but we can only speculate if the expression of *HoxB-6* in turtles starts anteriorly in the neck as seen in lizards or posteriorly in the trunk as seen in archosaurs and mammals.

A closer look at the cellular expression of *HoxC-6* has shown that it corresponds with the lack of ventral ribs in turtles^14^. In contrast to other amniotes, *HoxC-6* is expressed in the sclerotome (prevertebra) and the dermis in the epaxial domain (future carapacial dermis), but not in the somatopleure (embryonic region in which the ventral ribs are missing in turtles)^14^. It is assumed that the ventral ribs in turtles are likely to have been modified at least partly into the plastron^64, 65^. And there is evidence indicating that the plastron derivates from a late-emigrating population of trunk neural crest cells, which appears to be unique to turtles^65^. Although trunk neural crest cells are generally thought to lack skeletogenic potential (in contrast to cranial neural crest cells), these late-emigrating cells additionally contribute to the sclerotome-derived vertebral and rib cartilages in turtles^65^. The restoration of skeletogenic potential is assumed to be linked with loss of *Hox* gene expression^65, 66^.

In general, the expression of the *Hox* 7 genes is restricted to the dorsal vertebral column in amniotes (Fig. 5). *HoxA-7* starts at DV2 in crocodiles and chicken and at DV3 in mouse^13, 67, 68^. There is no information available for the lizard. In the turtle, however, the anterior expression limit of *HoxA-7* is in the neck at CV6^14^. Although possibly turtle-specific, the expression pattern of *HoxA-7* does not appear to be associated with the unique turtle body plan according to Ohya *et al*.^14^. These authors suggested that some of the differences in the *Hox* gene expression pattern in turtles may be neutral, both in terms of evolutionary changes and their developmental function^14^. It is striking to note that mouse *HoxA-7* mutants are healthy and display no abnormalities in the formation of the axial skeleton^69^. Nevertheless, in combination with a mutation in *HoxB-7* (double mutant) the functional role of *HoxA-7* in the patterning of the anterior dorsal vertebral region is evident^69^. This degree of functional redundancy between *HoxA-7* and *HoxB-7* opens the possibility that the former gene has been recruited for a turtle-specific function in contrast to Ohya *et al*.^14^ - equivalent to the recruitment of *HoxA-5* for rib repression in turtle and mouse as discussed above. I.e., turtles needed to evolve a specialized CV8 to articulate the highly mobile neck with the immobile trunk. This pattern is already recognizable in stem turtles. In pleurodires, the extent of lateral neck flexion remarkably increased compared to stem turtles – particularly in CV8^3^. As mentioned above, cryptodires evolved highly modified posterior cervical vertebrae to enable their highly derived retraction mode. This anatomical modification is also most likely related to the anterior *HoxA-7* expression.

The expression of the *Hox* genes of PG 8 is usually confined to the anterior or middle region of the dorsal vertebral column in amniotes (Fig. 5). The anterior expression limit of *HoxC-8* is at DV1 in crocodiles, at DV5 in the chicken, and at DV6 in the mouse^11–13^. There is no information available for the lizard. In the turtle, the expression of *HoxC-8* starts at DV3^14^. The anterior shift of H*oxC-8* expression in crocodiles relative to the other taxa has been interpreted to be a species-specific adaptation to enhance neural spine development of the dorsal vertebrae (i.e., larger dorsal neural spines supporting larger epaxial musculature)^13^. An originally crocodile-like *Hox* gene expression pattern in amniotes may have been modified in birds and mammals, which have undergone significant modifications in their axial skeleton^11^. Correspondingly, the expression of *HoxC-8* may be associated with the specialized body plan of turtles^14^. The study indicated that the anterior expression limit of *HoxC-8* at DV3 is consistent with the loss of the sternum and the modification of ventral ribs to parts of the plastron in turtles^14, 70^.

In summary, in contrast to archosaurs, at least one trunk-related *Hox* gene - *HoxA-7* - is involved in the formation of the cervical vertebral column in turtles. In this regard, the correlation between morphological modularity and *Hox* code in the neck of turtles differs from all other analyzed amniotes.

### Stem turtles, morphological modularity, and cervical ribs during turtle evolution

The present results indicate that the modular pattern of the Late Triassic stem turtle *Proganochelys quenstedti* is likely to represent the ancestral configuration for turtles because it is also present in the Early Cretaceous stem turtle *Naomichelys speciosa* (Fig. 4). The difference in morphological modularity in the neck of the Pleistocene turtle *Meiolania platyceps* may be explained by its distinct anatomy as a result of the long and separate evolution of this taxon^6^. Functional analyses, however, revealed that its short cervical vertebrae^71^ permit limited degrees of movement^72^. The presence of cervical ribs in *M. platyceps* may support the hypothesis of impairment of extreme movements in the neck^3^. And this may correspond to the relatively uniform modular pattern in the neck as indicated by the present analysis.

Cervical ribs appear to be plesiomorphically present in most basal turtles^36^. In living turtles, cervical ribs are highly rudimentary and evident only in embryos^7, 73^. The Miocene turtle *M. platyceps* has free ribs on CV2 to CV6 and the rib on CV7 is either absent as on CV8 or very small^37^ (Fig. 4). Although the cervical ribs are not preserved, the presence of free ribs on the cervical vertebrae of the Early Cretaceous stem turtle *N. speciosa* is assumed on basis of the presence of diapophyses and parapophyses on the vertebrae^74^. The Late Triassic stem turtle *P. quenstedti* has free ribs on CV2 to CV5 and the ribs appear to be fused on CV6 to CV8^39^ (Fig. 4). Considering the aforementioned rib-promoting and rib-suppressing role of *HoxA-5*, we, thus, hypothesize that the expression of *HoxA-5* was not yet shifted anteriorly in the stem turtles, but showed a more crocodilian-like posterior expression at the end of the neck and at CV8, respectively.

### Numerical constraint in the neck of turtles

Mammals are highly constrained in the number of cervical vertebrae (almost exclusively seven CV) and the neck kinematics rely on interspecific variation in vertebral morphology. Despite significant morphofunctional differences, the pattern of shape change within the neck appears to be consistent among diverse mammalian taxa^22, 23, 25, 26, 75^. Analogous to mammals with their “fixed” number of cervicals, the variable length of the neck in different turtle taxa arises from elongation or shortening of vertebrae because the number of cervical vertebrae is constant at eight. However, in contrast to mammals, in which the morphological regionalization within the neck appears to be similar across the species analyzed to date, turtles differ in the modular pattern of the cervical vertebral column. This difference appears to be associated with the specific neck retraction modes of cryptodiran and pleurodiran turtles, respectively, and specific modifications within these two groups. Correspondingly, the present study suggests that the underlying *Hox* code may show some variation. It would be relevant to reconstruct the anestral pattern to include in future studies stem turtles and relatives, such as the carapace-lacking *Odontochelys semitestacea* (eight cervicals), in order to reveal their modular pattern. This would be particularly interesting for taxa that differ from the turtle-specific vertebral count (eight CV, ten DV) since a gradual morphological transformation of DV1 to CV8 in Late Triassic turtles has been observed^18^.

**Figure 6:**
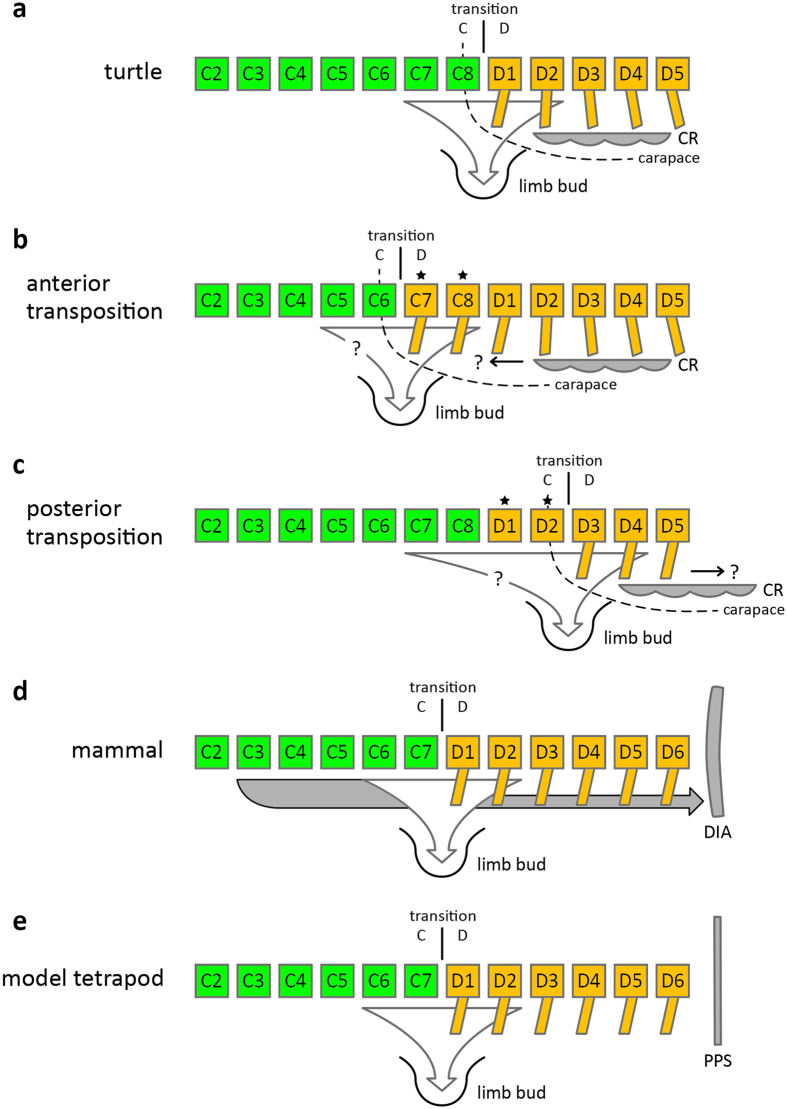
Numeric constraint in the cervical vertebral column in turtles. In general, the forelimb of tetrapods deviates from somitic cells of the cervical and dorsal (thoracic) vertebrae. An anterior or posterior transposition of the forelimb in relation to the axial skeleton can result in change of cervical count. However, this is postulated to be associated with adaptive costs and possibly affecting related anatomical structures (Buchholtz *et al*. 2012, Buchholtz 2014). (**a**) In turtles, the carapacial ridge (CR) forms posterior to the forelimb. It induces the fan-shape arrangement of the trunk ribs contributing to the development of the carapace. (**b** and **c**) An anterior or posterior transposition of the forelimb (more and less cervical vertebrae, respectively) may possibly affect the CR. This may generate a deficiency in the development of the turtle carapace. Asterisk indicates modified vertebral morphology. (**d**) In mammals, mid-cervical somitic cells migrate to form the diaphragm (DIA) (mammal based on Buchholtz *et al*. 2012, Buchholtz 2014). An anterior or posterior transposition of the forelimb are thought to generate a deficient diaphragm, forelimb, or both (Buchholtz *et al*. 2012, Buchholtz 2014). (**e**) In non-mammals, the post-pulmonary septum (PPS) is unmuscularized and an anterior or posterior transposition of the forelimb is unconstrained (model tetrapod based on Buchholtz *et al*. 2012, Buchholtz 2014).

Although there are different hypotheses, there is evidence suggesting that the numerical constraint in mammals may be the byproduct of the evolution of the muscularized diaphragm - a key innovation of mammals^22, 24^. The somites, from which the vertebrae arise during embryonic development, are also the source of the muscle cells of the diaphragm reviewed by Merrell & Kardon (2013)^76^. The muscle progenitors of the diaphragm migrate from the somites of CV3 to CV5^76^. Considering the origin of the cervical constraint in mammals, it has been hypothesized that both an anterior and posterior transposition of the forelimb (with less or more cervicals, respectively) are likely to generate a deficiency in the development of the muscularized diaphragm, in the forelimb, or in both^22, 24^ (Fig. 6). A key feature of the turtle ‘body plan’ is the carapace. It derives mainly from dorsal ribs that are laterally expanded through the axial arrest of turtle rib growth in combination with the development of the turtle-specific carapacial ridge (Fig. 6), a bulge at both sides of the trunk posterior to the limbs that induces the fan-shape arrangement of the ribs^77–84^. The evolutionary acquisition of the carapace is likely the product of the co-option of pre-existing, limb-bud related genes^85–87^. An anterior or posterior transposition of the forelimb can result in the change of cervical vertebral count, but may possibly affect the carapacial ridge. The anteroposterior movement of the limb may be constrained and, therefore, may potentially generate a deficiency in the development of the turtle carapace (Fig. 6). However, further morphogenetic studies are required to test this hypothetical scenario and to reveal if evolutionary novelties restrict flexibility in axial patterning in tetrapods.

## Conclusions

The present study provides a scenario of the evolution of axial patterning in Testudinata and indicates lineage-specific patterns based on a predictive model on *Hox* gene expression and vertebral shape correlation. The common modular pattern in the neck of stem turtles possibly represent the ancestral configuration for turtles. This pattern reveals similarities with that of archosaurs, which may hint at the phylogenetic relationship between archosaurs and turtles. In this regard, further morphological analyses including important key taxa may help provide new insights. An important modification that is likely to have occurred during the evolution towards modern turtles is the anterior shift of *HoxA-5* expression and of *HoxB-5*. The former is linked to the reduction of cervical ribs. The latter is associated with a lower morphological differentiation between the anterior cervical vertebrae.

The comparison of the morphological modularity between the analyzed Pleurodira and Cryptodira indicates that the middle region of the neck in pleurodiran turtles is more uniform. This corresponds to their lateral neck retraction mode which is linked to highly specialized mid-vertebrae. In contrast, the modular pattern in cryptodiran turtles revealed a higher degree of morphological differentiation. The vertical neck retraction mode of these species is associated with a highly specialized posterior cervical vertebral region, which is related to the unique neck-trunk articulation. It would be highly important to directly study the underlying genetic differences of the morphological modularity between Pleurodira and Cryptodira. It may be possible that the anterior shift of *HoxA-7* expression in the neck of *Pelodiscus sinensis* is a cryptodiran-specific feature related to their unique CV8/DV1 articulation but, as mentioned above, also pleurodires show a separated morphometric module of CV8, which is possibly related to their derived mode of lateral neck flexion and also influenced by *HoxA-7* expression.

Finally, the modular pattern in the neck of the analyzed turtles supports the hypothesis that the *Hox* code reflects their unique body plan. Modifications in *Hox* gene patterning are again shown to be crucial to understand evolutionary novelties. Further genetic studies are required in order to improve our knowledge of the *Hox* gene expression pattern of living turtles. Interestingly, turtles display a high level of constraint in cervical vertebral count as it has been noted for mammals^16^. Due to the species-specific neck retraction modes, the numerical constraint is not linked with a common *Hox* code in the cervical vertebral column as suggested for mammals^25^. In a morphogenetic triad, shell, neck, and head morphology influence each other^2^. Our analysis shows that neck development strongly mediates between the components of unique ‘bauplan’ of turtles. Although our reconstructions provide a consistent picture on the evolution of the cervical vertebral column in turtles, more genes and gene networks beyond *Hox* patterning need to be included in future analyses to analyze the exceptional turtle anatomy.

## Electronic supplementary material

Supplementary Information

## References

[1] Laurin, M. *How Vertebrates Left The Water*. 216 (University of California Press, 2010).

[2] Werneburg I (2015). Neck motion in turtles and its relation to the shape of the temporal skull region. Comptes Rendus Palevol..

[3] Werneburg I (2015). Modeling neck mobility in fossil turtles. J. Exp. Zool. B Mol. Dev. Evol..

[4] Gaffney ES (1975). A phylogeny and classification of the higher categories of turtles. Bull. Am. Mus. Nat. Hist..

[5] Herrel, A., Van Damme, J. & Aerts, P. In *Biology of Turtles: From Structures to Strategies of Life* (eds J. Wyneken, M. H. Godfrey, & V. Bels) 163–212 (CRC Press Taylor & Francis Group, 2008).

[6] Werneburg I, Wilson LA, Parr WC, Joyce WG (2015). Evolution of neck vertebral shape and neck retraction at the transition to modern turtles: an integrated geometric morphometric approach. Syst. Biol..

[7] Werneburg I, Maier W, Joyce WG (2013). Embryonic remnants of intercentra and cervical ribs in turtles. Biol. Open.

[8] Williams EE (1950). Variation and selection in the cervical central articulations of living turtles. Bull. Am. Mus. Nat. Hist..

[9] Dalrymple GH (1979). Packaging problems of head retraction in trionychid turtles. Copeia.

[10] Van Damme J, Aerts P, De Vree F (1995). Kinematics of the escape head retraction in the common snake-necked turtle *Chelodina longicollis* (Testudines: Pleurodira: Chelidae). Belg. J. Zool..

[11] Böhmer C, Rauhut OWM, Wörheide G (2015). New insights into the vertebral Hox code of archosaurs. Evol. Dev.

[12] Burke AC, Nelson CE, Morgan BA, Tabin C (1995). *Hox* genes and the evolution of vertebrate axial morphology. Development.

[13] Mansfield JH, Abzhanov A (2010). *Hox* expression in the American alligator and evolution of archosaurian axial patterning. J. Exp. Zool. B Mol. Dev. Evol..

[14] Ohya YK, Kuraku S, Kuratani S (2005). *Hox* code in embryos of Chinese soft-shelled turtle *Pelodiscus sinensis* correlates with the evolutionary innovation in the turtle. J. Exp. Zool. B Mol. Dev. Evol..

[15] Böhmer, C., Rauhut, O. W. M. & Wörheide, G. Correlation between *Hox* code and vertebral morphology in archosaurs. *Proc. Roy. Soc. Lond. B Biol. Sci*. **282**, doi:10.1098/rspb.2015.0077 (2015). 10.1098/rspb.2015.0077 PMC4590465 26085583

[16] Müller J (2010). Homeotic effects, somitogenesis and the evolution of vertebral numbers in recent and fossil amniotes. Proc. Natl. Acad. Sci. USA.

[17] Li C, Wu XC, Rieppel O, Wang LT, Zhao LJ (2008). An ancestral turtle from the Late Triassic of southwestern China. Nature.

[18] Szczygielski T (2017). Homeotic shift at the dawn of the turtle evolution. Roy. Soc. Open Sci..

[19] Sánchez-Villagra MR, Narita Y, Kuratani S (2007). Thoracolumbar vertebral number: the first skeletal synapomorphy for afrotherian mammals. Syst. Biodivers..

[20] Galliari FC, Carlini AA, Sánchez-Villagra MR (2010). Evolution of the axial skeleton in armadillos (Mammalia, Dasypodidae). Mamm. Biol..

[21] Narita Y, Kuratani S (2005). Evolution of the vertebral formulae in mammals: a perspective on developmental constraints. J. Exp. Zool. B Mol. Dev. Evol..

[22] Buchholtz EA (2012). Fixed cervical count and the origin of the mammalian diaphragm. Evol. Dev..

[23] Johnson DR, McAndrew TJ, Oguz Ö (1999). Shape differences in the cervical and upper thoraic vertebrae in rats (*Rattus norvegicus*) and bats (*Pteropus poiocephalus*): can we see shape patterns derived from position in column and species membership?. J. Anat..

[24] Buchholtz EA (2014). Crossing the frontier: a hypothesis for the origins of meristic constraint in mammalian axial patterning. Zoology.

[25] Böhmer C (2017). Correlation between *Hox* code and vertebral morphology in the mouse: towards a universal model for Synapsida. Zoological Lett..

[26] Arnold P, Forterre F, Lang J, Fischer MS (2016). Morphological disparity, conservatism, and integration in the canine lower cervical spine: insights into mammalian neck function and regionalization. Mamm. Biol..

[27] Varela-Lasheras I (2011). Breaking evolutionary and pleiotropic constraints in mammals: On sloths, manatees and homeotic mutations. EvoDevo.

[28] Head JJ, Polly PD (2015). Evolution of the snake body form reveals homoplasy in amniote Hox gene function. Nature.

[29] Harrell, F. E. Jr. Regression Modeling Strategies. With Applications to Linear Models, Logistic Regression, and Survival Analysis. (Springer, 2001).

[30] Landmark v. 3.0 (Institute for Data Analysis and Visualization (IDAV), University of California, Davis, 2005).

[31] Hammer Ø, Harper DAT, Ryan PD (2001). PAST: Palaeontological Statistics software package for education and data analysis. Palaeontol. Electron..

[32] Liang D, Wu R, Geng J, Wang C, Zhang P (2011). A general scenario of Hox gene inventory variation among major sarcopterygian lineages. BMC Evol. Biol..

[33] Kessel M, Gruss P (1990). Murine developmental control genes. Science.

[34] Nixon KC, Carpenter JM (2012). On homology. Cladistics.

[35] Witmer, L. M. In *Functional morphology in vertebrate paleontology* (ed J. Thomason) Ch. 2, 19–33 (Cambridge University Press, 1995).

[36] Joyce, W. G. Phylogenetic relationships of Mesozoic turtles. *Bull. Peabody Mus. Nat. Hist*. **48** (2007).

[37] Gaffney ES (1985). The cervical and caudal vertebrae of the cryptodiran turtle, Meiolania platyceps, from the Pleistocene of Lord Howe Island, Australia. Am. Mus. Novit.

[38] Sterli J, de la Fuente S (2011). A new turtle from the La Colonia Formation (Campanian-Maastrichtian), Patagonia, Argentina, with remarks on the evolution of the vertebral column in turtles. Palaeontology.

[39] Gaffney ES (1990). The comparative osteology of the Triassic turtle. Proganochelys. Bull. Am. Mus. Nat. Hist.

[40] Mallo M, Wellik DM, Deschamps J (2010). *Hox* genes and regional patterning of vertebrate body plan. Dev. Biol..

[41] Gaunt SJ (1994). Conservation in the *Hox* code during morphological evolution. Int. J. Dev. Biol..

[42] Amemiya CT (2010). Complete HOX cluster characterization of the coelacanth provides further evidence for slow evolution of its genome. Proc. Natl. Acad. Sci. USA.

[43] Prince VE, Joly L, Ekker M, Ho RK (1998). Zebrafish *hox* genes: genomic organization and modified colinear expression patterns in the trunk. Development.

[44] Wellik DM, Capecchi MR (2003). *Hox10* and *Hox11* genes are required to globally pattern the mammalian skeleton. Science.

[45] McIntyre DC (2007). Hox patterning of the vertebrate rib cage. Development.

[46] Yekta S, Tabin CJ, Bartel DP (2008). MicroRNAs in the *Hox* network: an apparent link to posterior prevalence. Nat. Rev. Genet..

[47] Kostic D, Capecchi MR (1994). Targeted disruptions of the murine *Hoxa-4* and *Hoxa-6* genes result in homeotic transformations of components of the vertebral column. Mech. Dev..

[48] Aubin J, Lemieux M, Tremblay M, Behringer RR, Jeannotte L (1998). Transcriptional interferences at the *Hoxa4/Hoxa5* locus: importance of correct *Hoxa5* expression for the proper specification of the axial skeleton. Dev. Dyn..

[49] Chen JW (2013). Hoxa-5 acts in segmented somites to regulate cervical vertebral morphology. Mech. Dev..

[50] Horan GSB, Kovàcs EN, Behringer RR, Featherstone MS (1995). Mutations in paralogous *Hox* genes result in overlapping homeotic transformations of the axial skeleton: evidence for unique and redundant function. Dev. Biol..

[51] Rancourt DE, Tsuzuki T, Capecchi MR (1995). Genetic interaction between hoxb-5 and hoxb-6 is revealed by nonallelic noncomplementation. Genes Dev..

[52] Huang R, Zhi Q, Patel K, Wilting J, Christ B (2000). Contribution of single somites to the skeleton and muscles of the occipital and cervical regions in avian embryos. Anat. Embryol..

[53] Woltering JM (2009). Axial patterning in snakes and caecilians: evidence for an alternative interpretation of the *Hox* code. Dev. Biol..

[54] Ogushi K (1911). Anatomische Studien an der japanischen dreikralligen Lippenschildkröte (*Trionyx japanicus*). I. Mitteilung. Morphol. Jahrb..

[55] Sanchez-Villagra MR (2009). Skeletal development in the Chinese soft-shelled turtle *Pelodiscus sinensis* (Testudines: Trionychidae). J. Morphol..

[56] Nagashima H (2009). Evolution of the turtle body plan by the folding and creation of new muscle connections. Science.

[57] Lyson TR, Joyce WG (2012). Evolution of the turtle bauplan: the topological relationship of the scapula relative to the ribcage. Biol. Lett..

[58] Bojanus, L. H. *Anatome testudinis europaeae (An anatomy of the turtle)*. (Society for the Study of Amphibians and Reptiles, 1819).

[59] Gaunt SJ, Krumlauf R, Duboule D (1989). Mouse homeo-genes within a subfamily, Hox-1.4, -2.6 and -5.1, display similar anteroposterior domains of expression in the embryo, but show stage- and tissue-dependent differences in their regulation. Development.

[60] Jeannotte L, Lemieux M, Charron J, Poirier F, Robertson EJ (1993). Specification of axial identity in the mouse: role of the Hoxa-5 (Hox1.3) gene. Genes Dev..

[61] Toth LE, Slawin KL, Pintar JE, Nguyen-Huu MC (1987). Region-specific expression of mouse homeobox genes in the embryonic mesoderm and central nervous system. Proc. Natl. Acad. Sci. USA.

[62] Nowicki JL, Burke AC (2000). *Hox* genes and morphological identity: axial versus lateral patterning in the vertebrate mesoderm. Development.

[63] Williams EE (1959). Cervical ribs in turtles. Breviora Museum of Comparative Zoology.

[64] Rice R, Kallonen A, Cebra-Thomas J, Gilbert SF (2016). Development of the turtle plastron, the order-defining skeletal structure. Proc. Natl. Acad. Sci. USA.

[65] Cebra-Thomas JA (2007). Evidence that a late-emerging population of trunk neural crest cells forms the plastron bones in the turtle Trachemys scripta. Evol. Dev..

[66] McGonnell IM, Graham A (2002). Trunk neural crest has skeletogenic potential. Curr. Biol..

[67] Puschel AW, Balling R, Gruss P (1990). Position-specific activity of the Hox1.1 promoter in transgenic mice. Development.

[68] Gaunt SJ, Dean W, Sang H, Burton RD (1999). Evidence that *Hoxa* expression domains are evolutionarily transposed in spinal ganglia, and are established by forward spreading in paraxial mesoderm. Mech. Dev..

[69] Chen F, Greer J, Capecchi MR (1998). Analysis of Hoxa7/Hoxb7 mutants suggests periodicity in the generation of the different sets of vertebrae. Mech. Dev..

[70] Schoch RR, Sues HD (2015). A Middle Triassic stem-turtle and the evolution of the turtle body plan. Nature.

[71] Gaffney ES (1996). The postcranial morphology of *Meiolania platyceps* and a review of of the Meiolaniidae. Bull. Am. Mus. Nat Hist..

[72] Jannel, A. Neck mobility, grazing habits, and intraspecific combat behaviour in the Giant Pleistocene horned turtle *Meiolania platyceps*. Master thesis, Uppsala University (2015).

[73] Williams EE (1959). Gadow’s arcualia and the development of tetrapod vertebrae. Q. Rev. Biol..

[74] Joyce WG, Sterli J, Chapman SD (2014). The skeletal morphology of the solemydid turtle *Naomichelys speciosa* from the early Cretaceous of Texas. J. Paleontol..

[75] O’Higgins P, Milne N, Johnson DR, Runnion CK, Oxnard CE (1997). Adaptation in the vertebral column: a comparative study of patterns of metameric variation in mice and men. J. Anat..

[76] Merrell AJ, Kardon G (2013). Development of the diaphragm - a skeletal muscle essential for mammalian respiration. FEBS Journal.

[77] Burke AC (1989). Development of the turtle carapace: Implications for the evolution of a novel bauplan. J. Morphol..

[78] Nagashima H (2007). On the carapacial ridge in turtle embryos: its developmental origin, function and the chelonian body plan. Development.

[79] Pascual-Anaya J, Hirasawa T, Sato I, Kuraku S, Kuratani S (2014). Comparative analysis of pleurodiran and cryptodiran turtle embryos depicts the molecular ground pattern of the turtle carapacial ridge. Int. J. Dev. Biol..

[80] Hirasawa T, Nagashima H, Kuratani S (2013). The endoskeletal origin of the turtle carapace. Nat. Commun..

[81] Hirasawa T (2015). The evolutionary origin of the turtle shell and its dependence on the axial arrest of the embryonic rib cage. J. Exp. Zool. B Mol. Dev. Evol..

[82] Werneburg I, Sanchez-Villagra MR (2009). Timing of organogenesis support basal position of turtles in the amniote tree of life. BMC Evol. Biol..

[83] Werneburg I (2009). A Standard System to Study Vertebrate Embryos. PLoS ONE.

[84] Werneburg I, Hugi J, Muller J, Sanchez-Villagra MR (2009). Embryogenesis and ossification of Emydura subglobosa (Testudines, Pleurodira, Chelidae) and patterns of turtle development. Dev. Dyn..

[85] Kuraku S, Usuda R, Kuratani S (2005). Comprehensive survey of carapacial ridge-specific genes in turtle implies co-option of some regulatory genes in carapace evolution. Evol. Dev..

[86] Moustakas JE (2008). Development of the carapacial ridge: implications for the evolution of genetic networks in turtle shell development. Evol. Dev..

[87] Joyce WG, Parham JF, Lyson TR, Warnock RCM, Donoghue PCJ (2013). A divergence dating analysis of turtles using fossil calibrations: an example of best practice. J. Paleontol..

