# Supplementary material for: Deep time perspective on turtle neck evolution: chasing the Hox code by vertebral morphology
Source: Sci Rep. 2017 Aug 21;7:8939. doi: 10.1038/s41598-017-09133-0 (PMC5566328; doi:10.1038/s41598-017-09133-0)
Supplement: Supplementary file 1 — Supplementary Information [file 41598_2017_9133_MOESM1_ESM.doc]

**Supplementary Material**

*Titel:* **Deep time perspective on turtle neck evolution: chasing the *Hox* code by vertebral morphology**

*Authors, email addresses:*

Christine Böhmer*^1^ boehmer@vertevo.de

Ingmar Werneburg*^2,3,4^ ingmar.werneburg@senckenberg.de

*Institutional addresses:*

^1^UMR 7179 CNRS/MNHN, Muséum National d’Histoire Naturelle, 57 rue Cuvier CP-55, 75005 Paris, France

^2^Senckenberg Center for Human Evolution and Palaeoenvironment at Eberhard Karls Universität, Sigwartstr. 10, 72076 Tübingen, Germany

^3^Fachbereich Geowissenschaften, Eberhard Karls Universität, Hölderlinstraße 12, D-72074 Tübingen, Germany

^4^Museum für Naturkunde, Leibniz-Institut für Evolutions- und Biodiversitätsforschung an der Humboldt-Universität zu Berlin, Invalidenstraße 43, 10115 Berlin, Germany

**Corresponding authors:*

Christine Böhmer, Ingmar Werneburg

**Supplementary figures**

**Figure S1**: Relative Warps (RW) analysis result (plot of first two RW; x-axis = RW1, y-axis = RW2) and cluster analysis results for all analyzed taxa.

**A** *Proganochelys quenstedti*


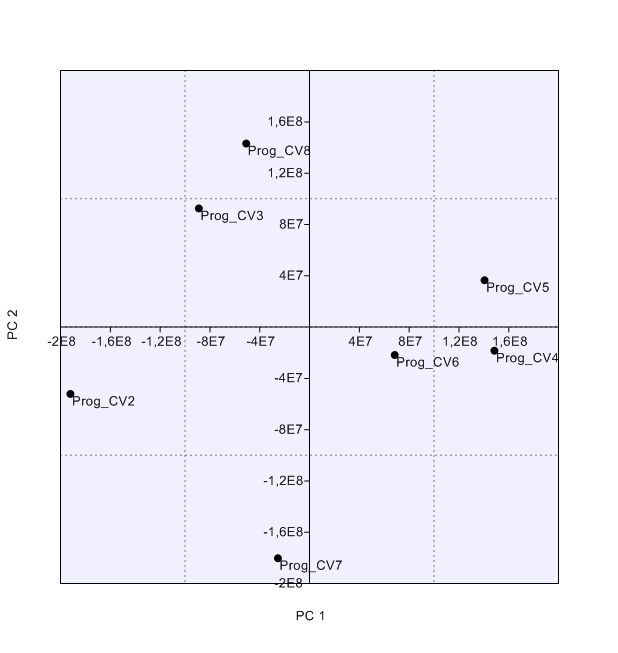


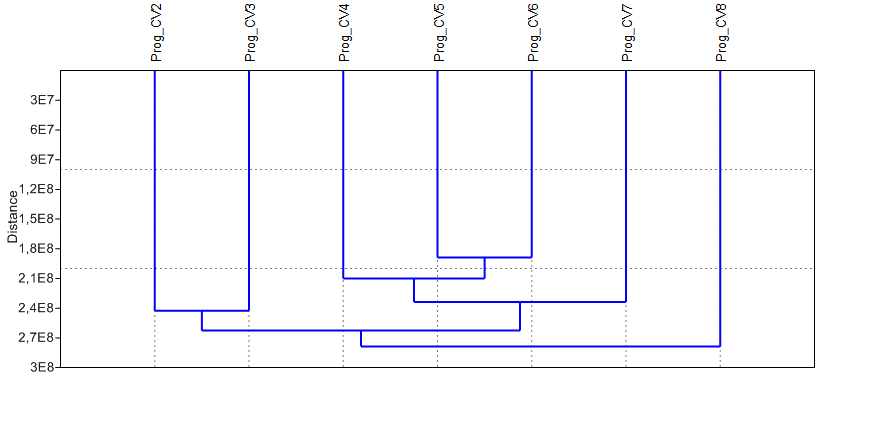


**B** *Meiolania* *platyceps*


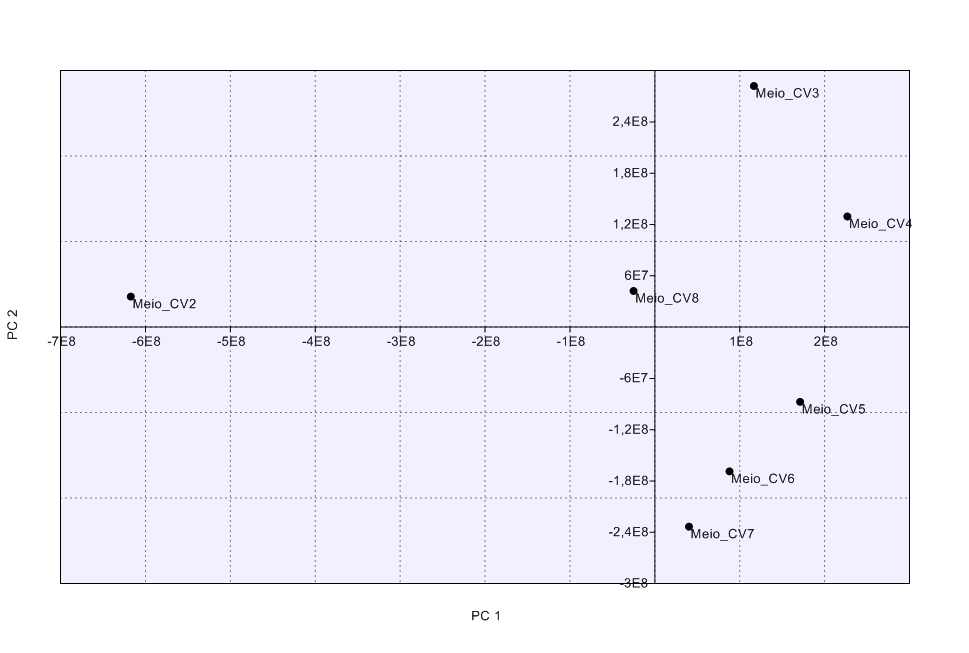


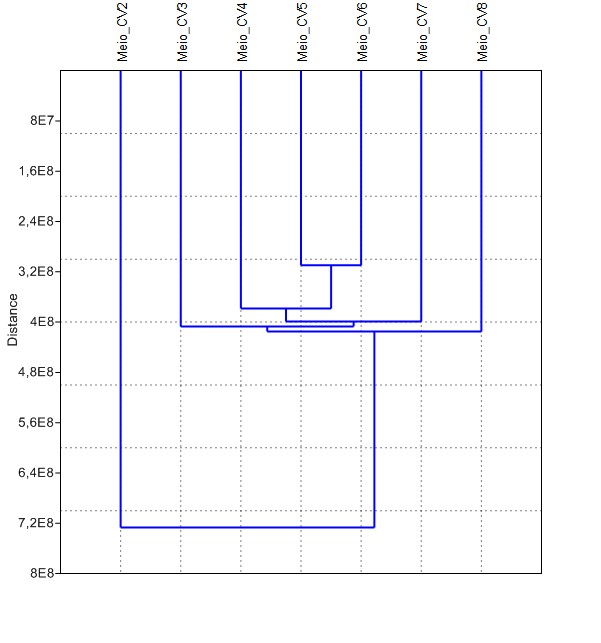


**C** *Naomichelys speciosa*


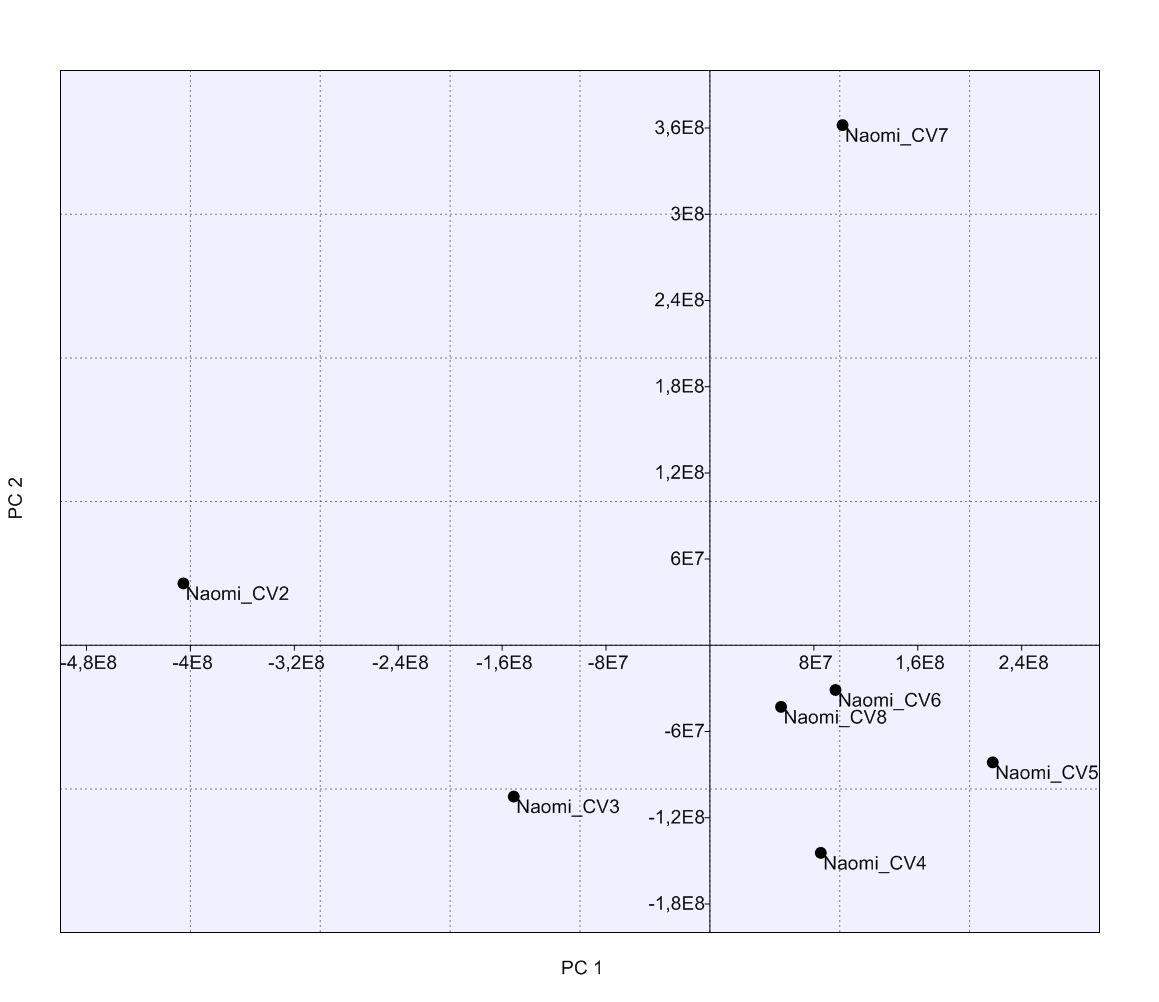


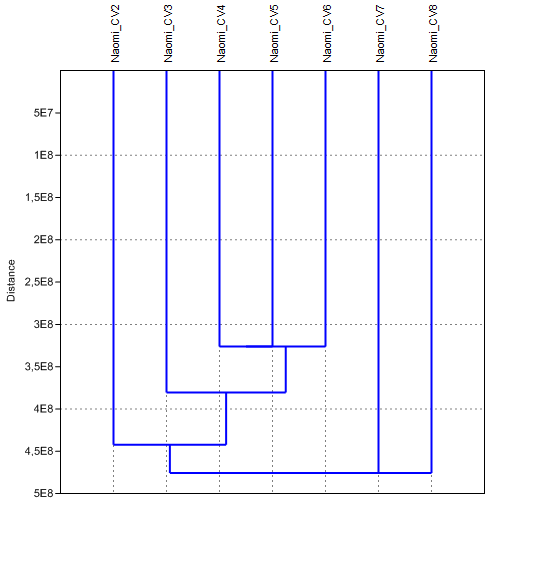


**D** *Hydromedusa tectifera*


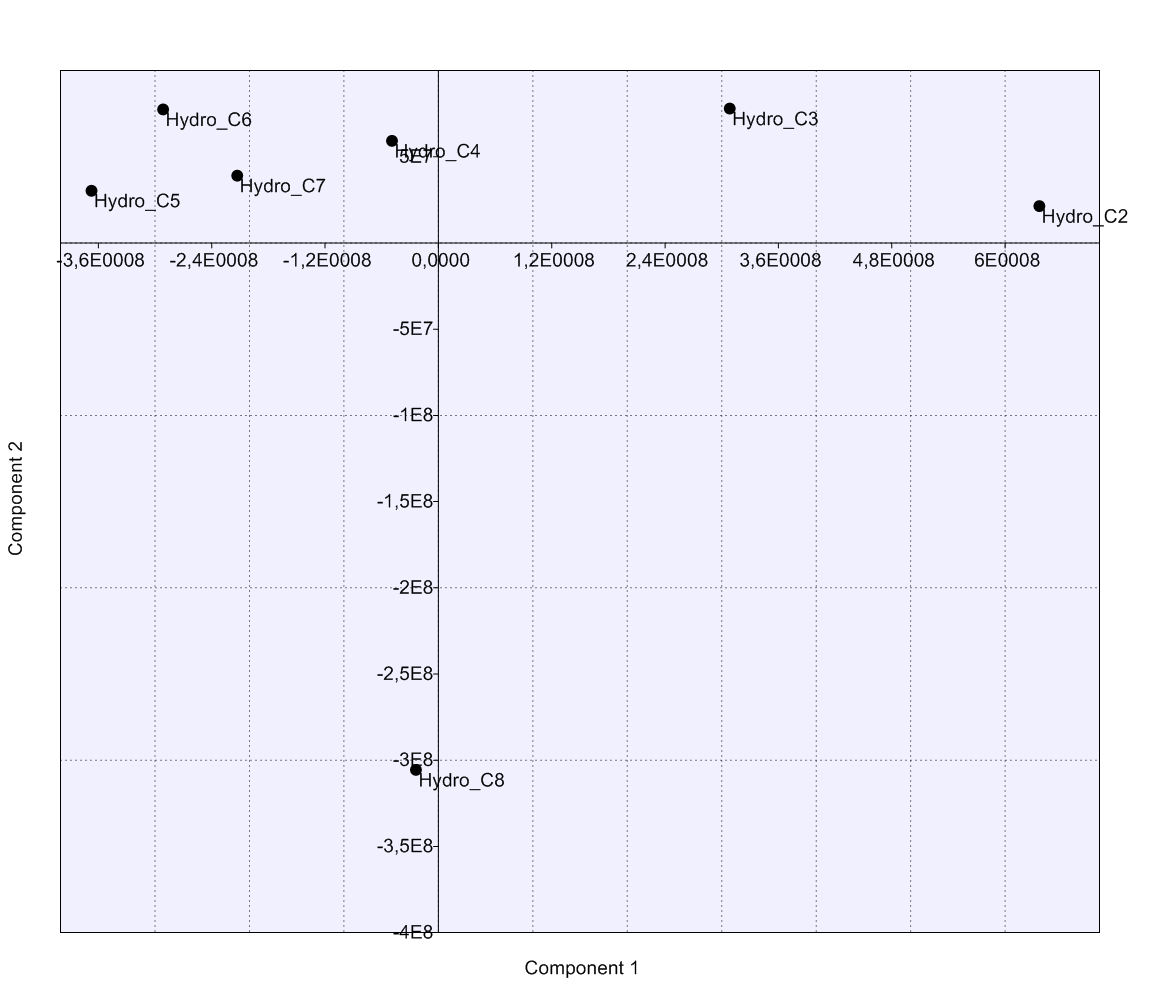


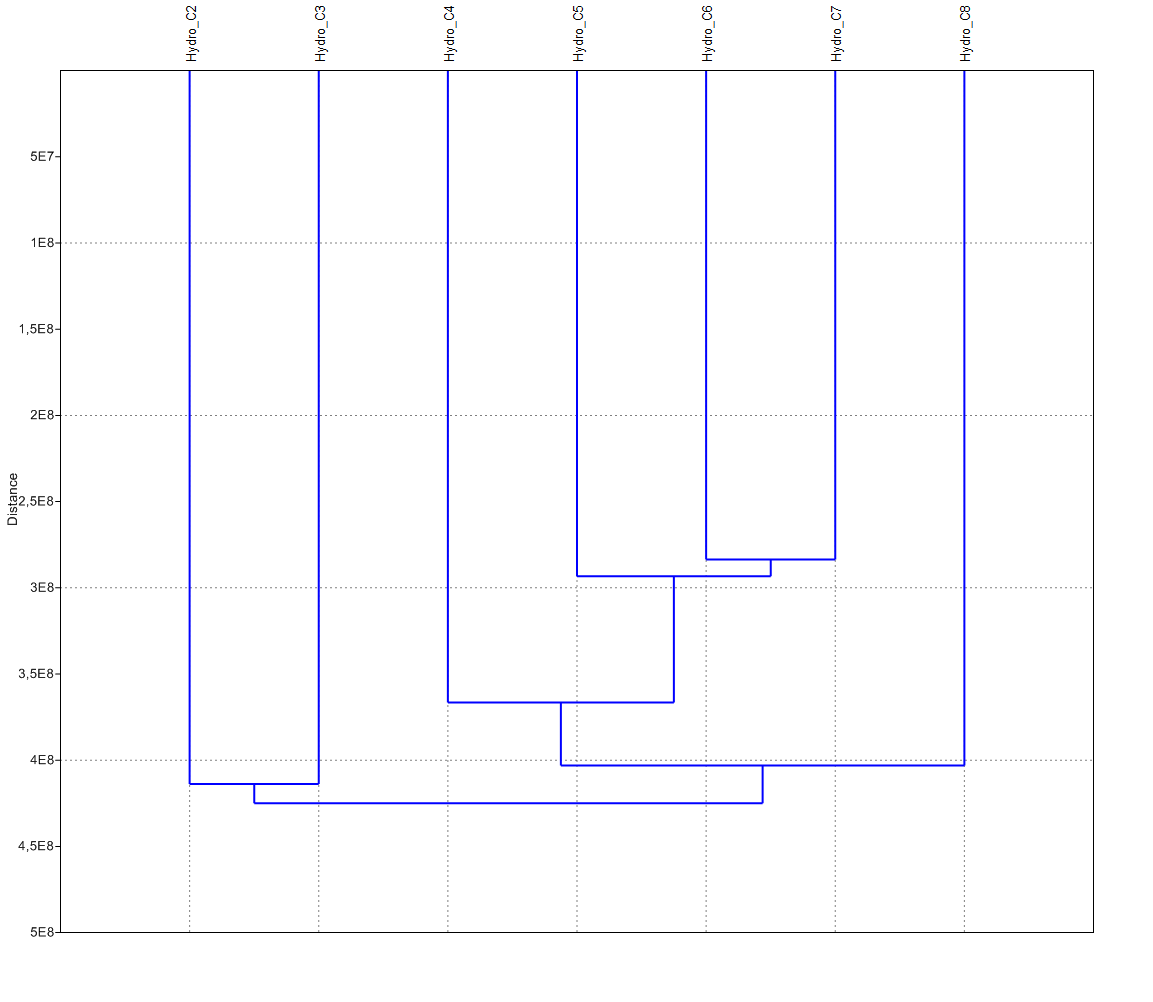


**E** *Phrynops geoffroanus*


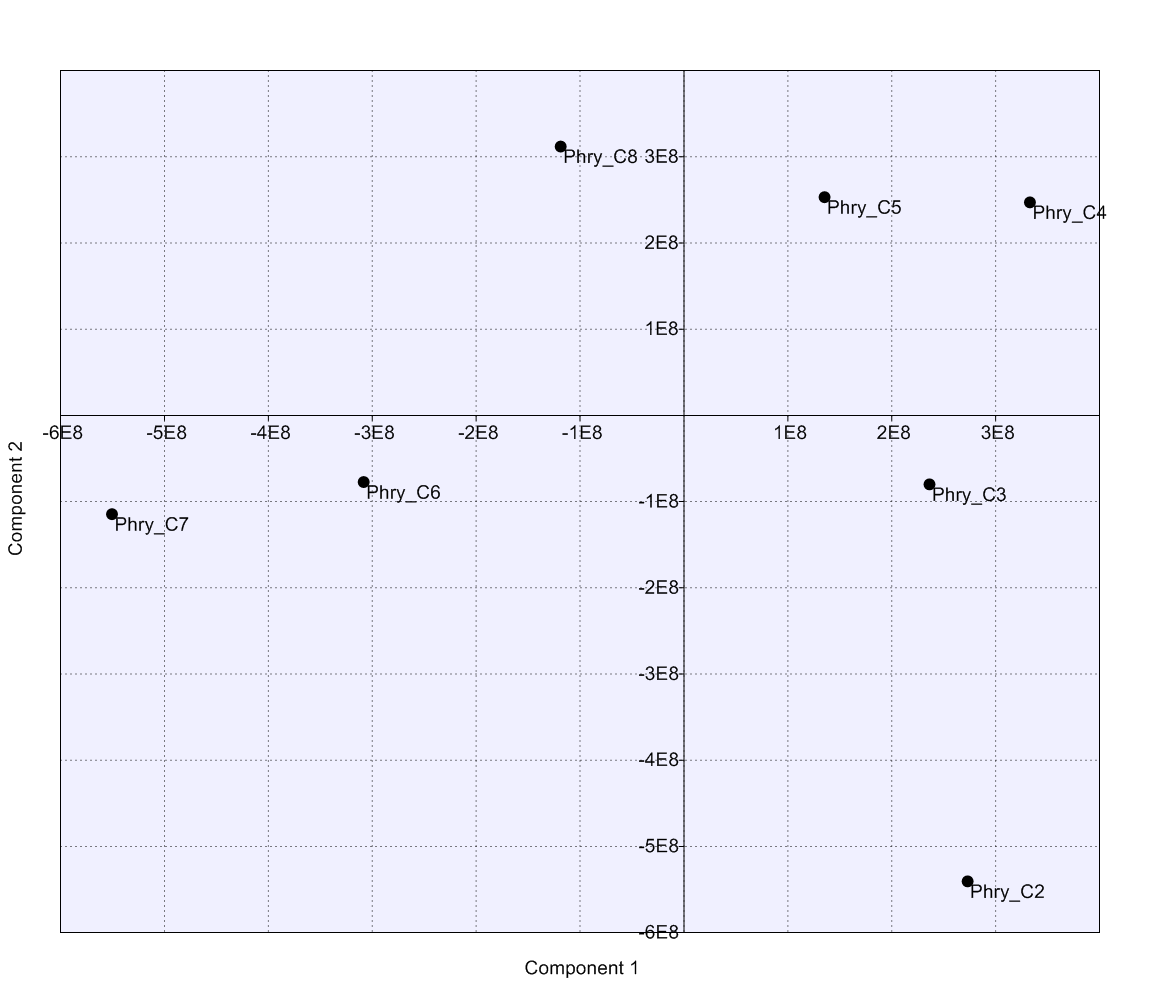


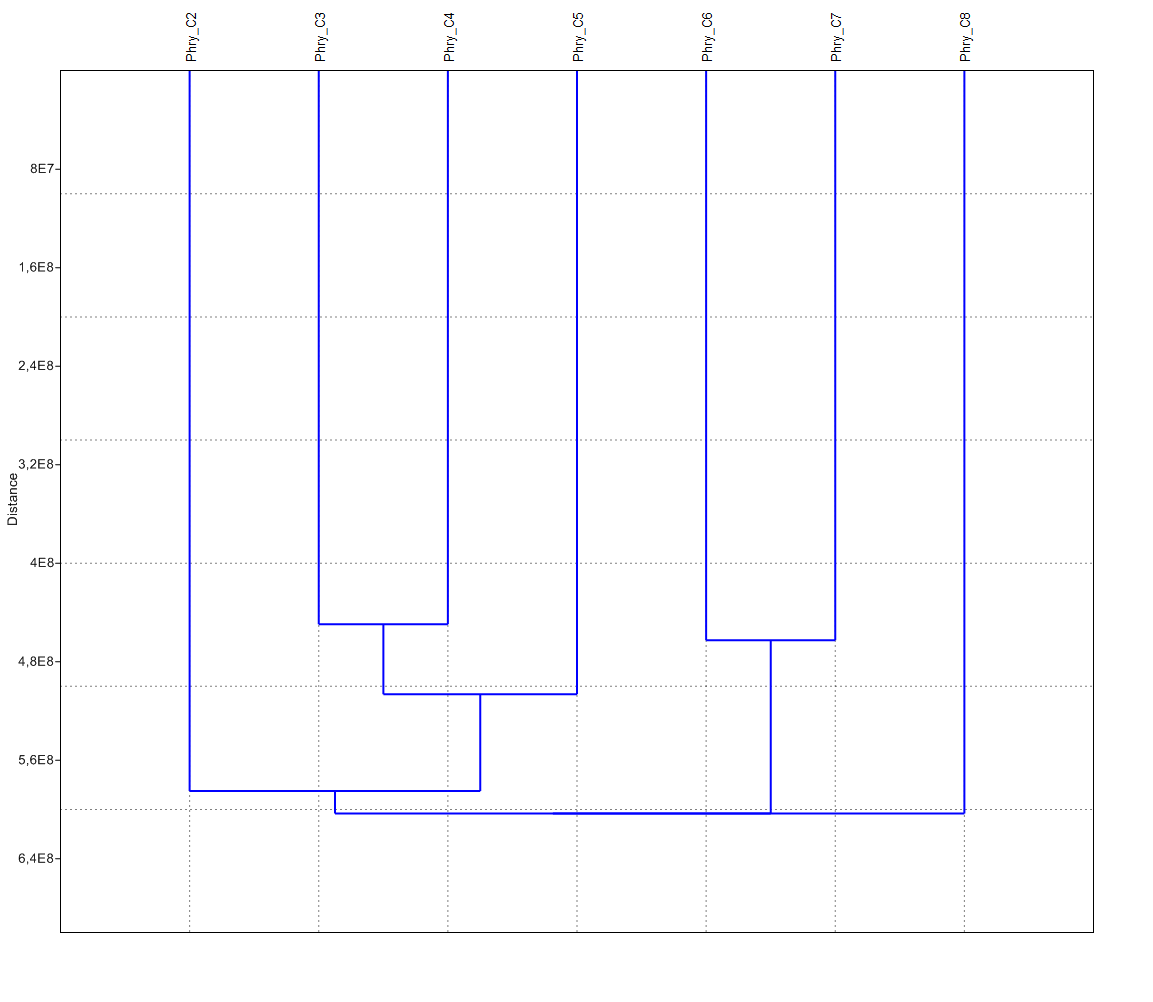


**F** *Macrochelys temmincki*


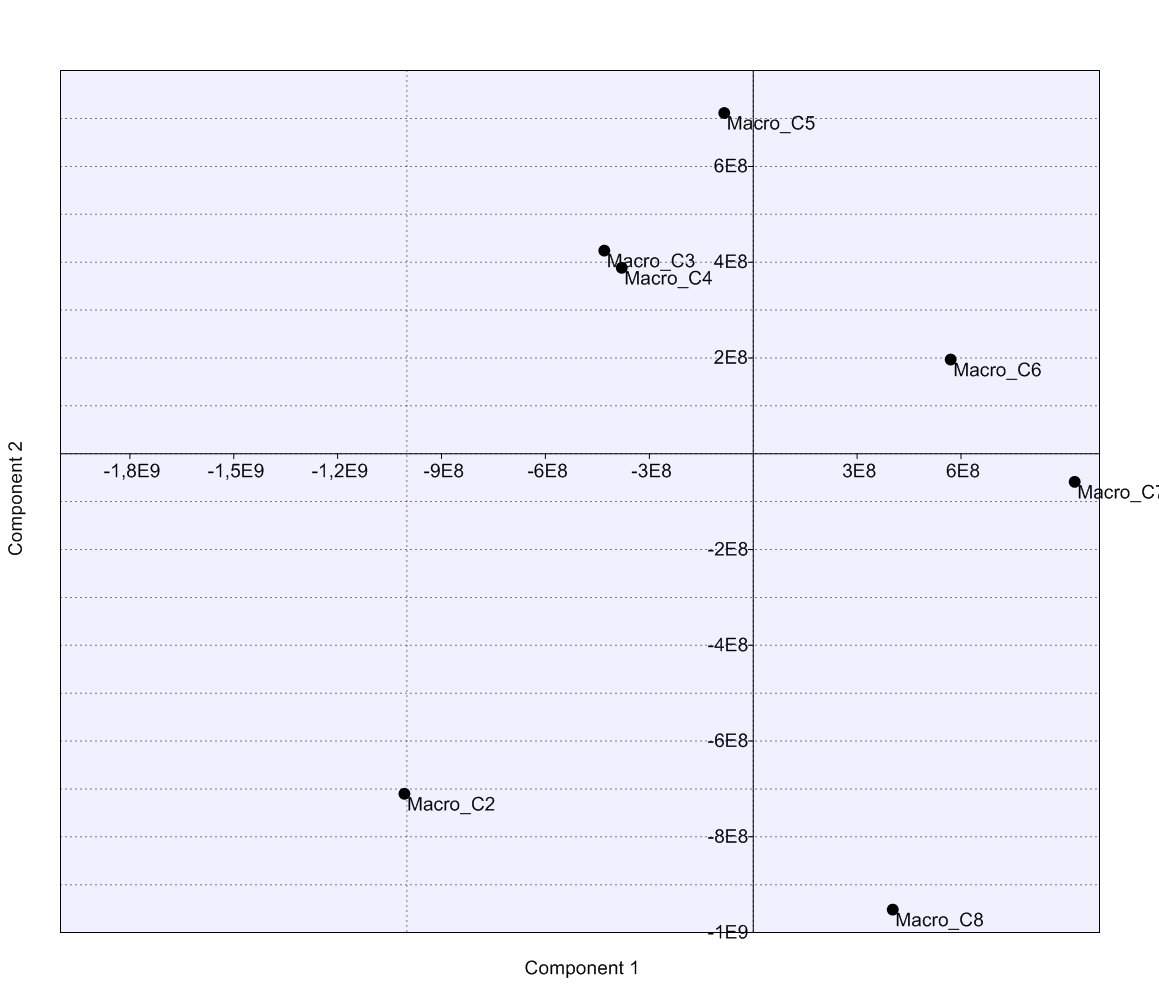


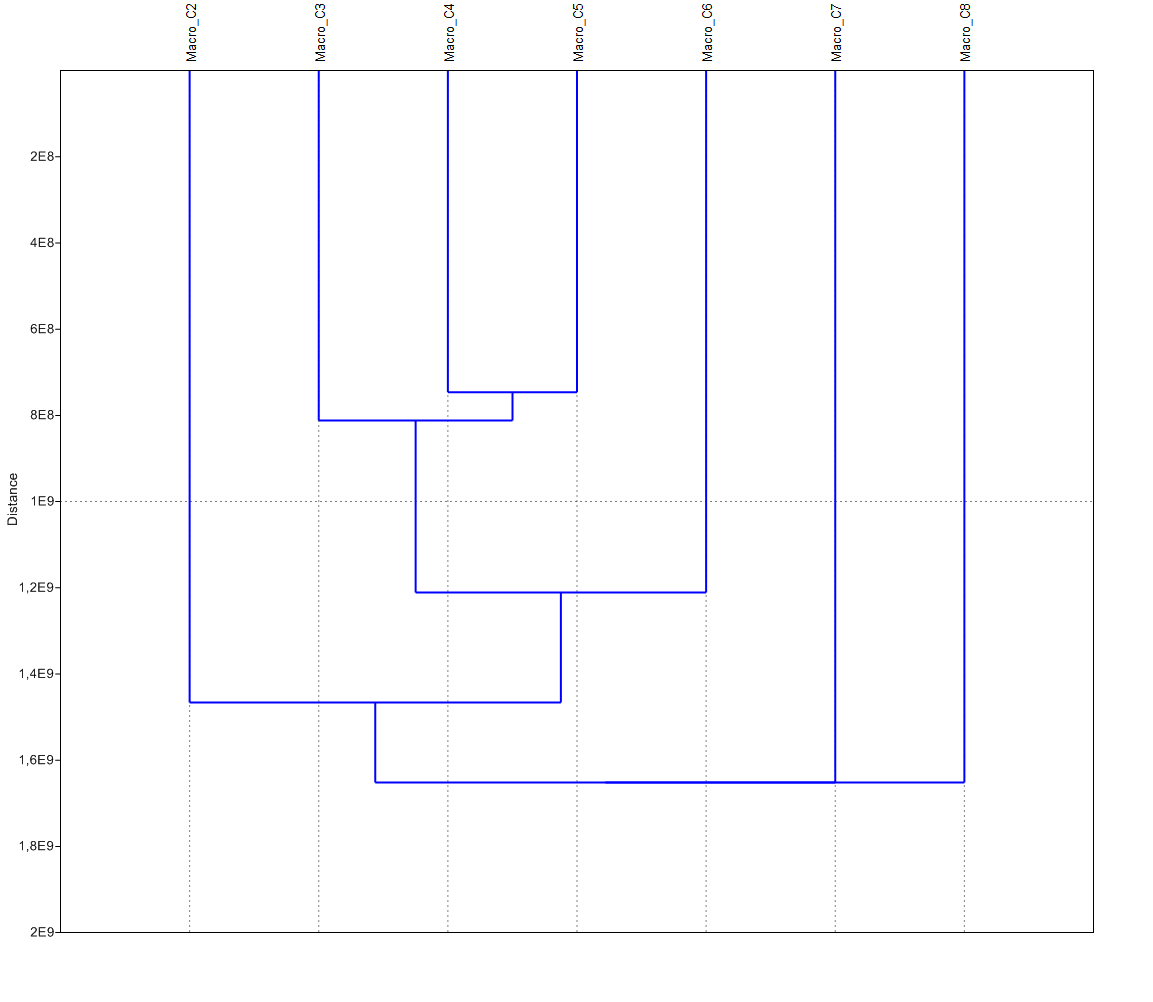


**G** *Kinosternon scorpioides*


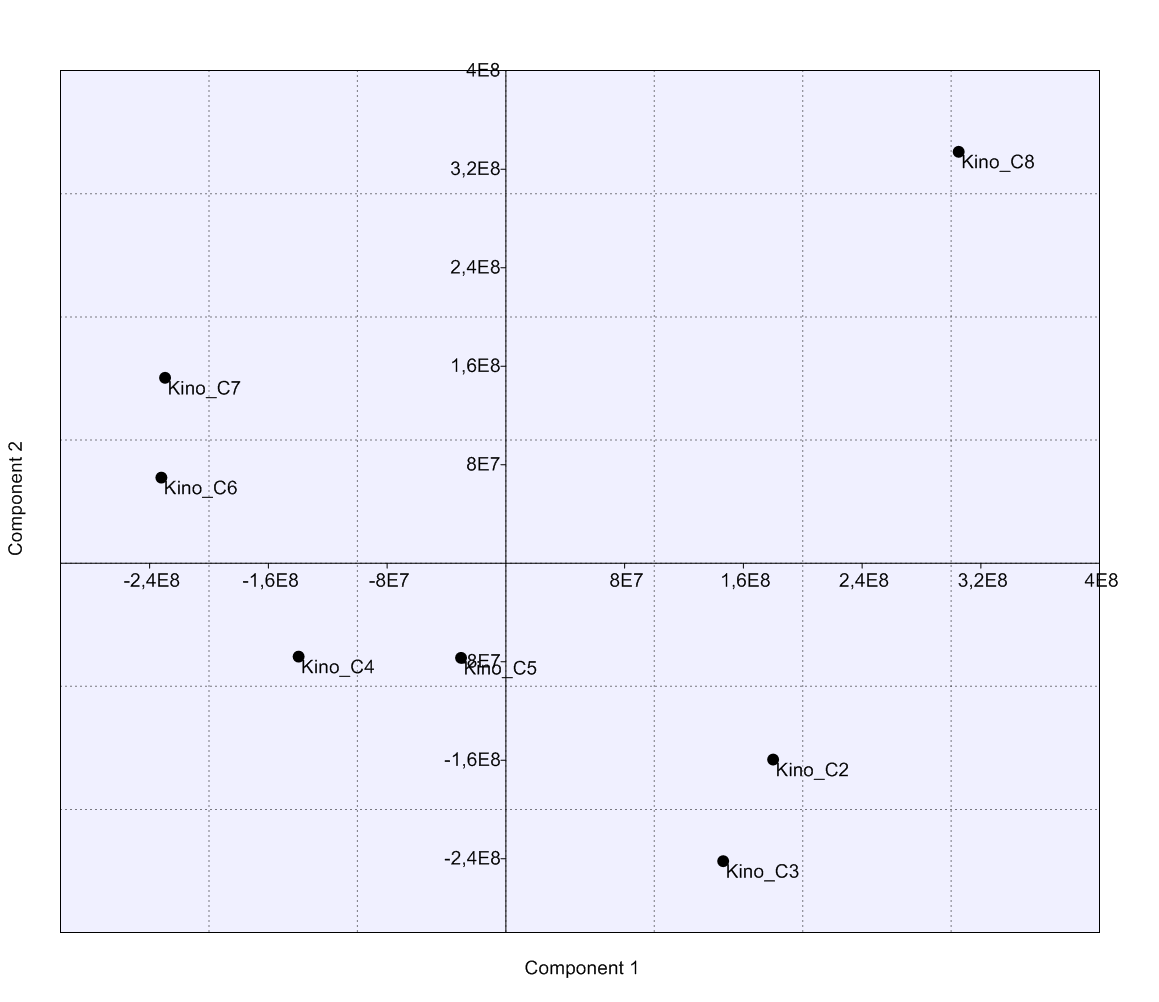


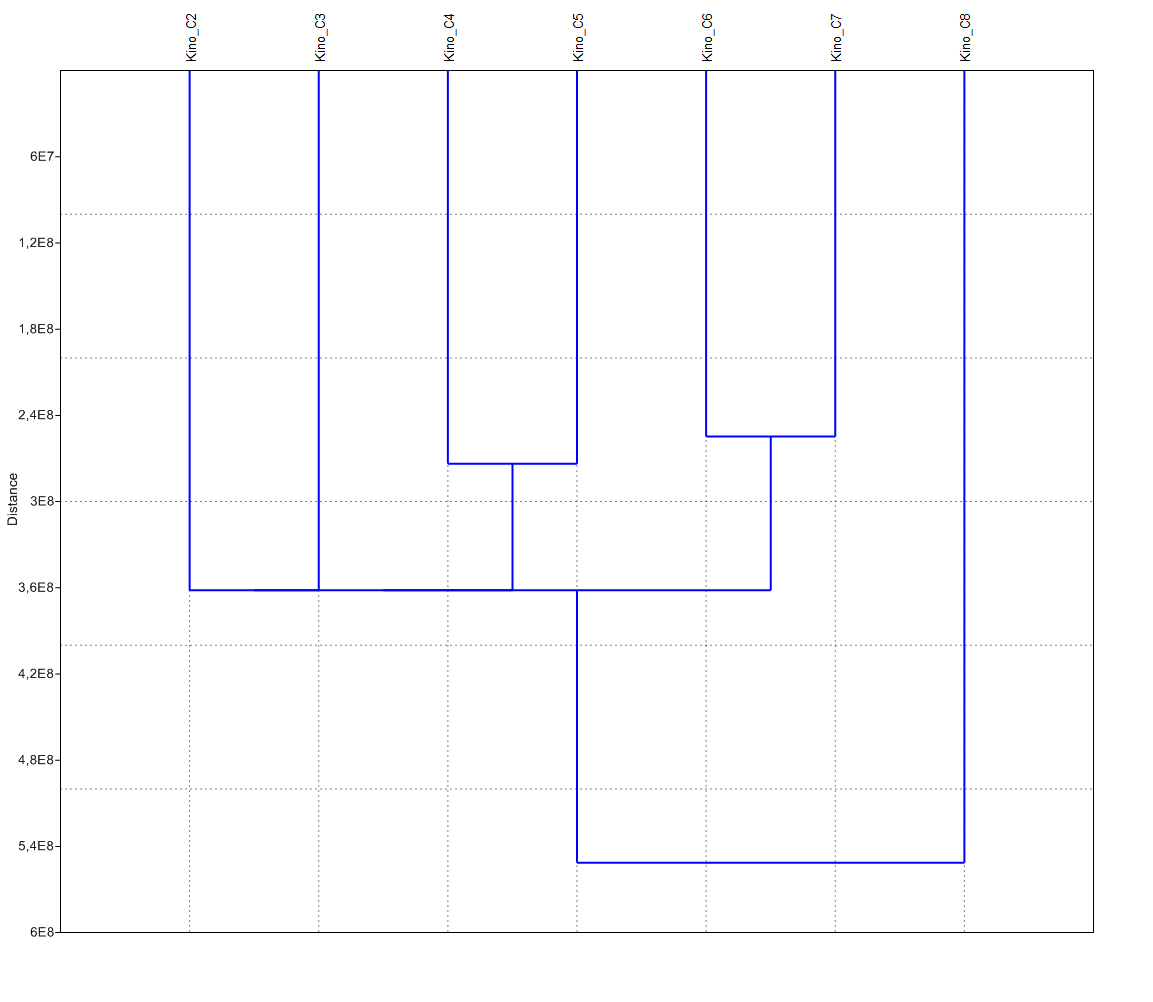


**H** *Dermatemys mawii*


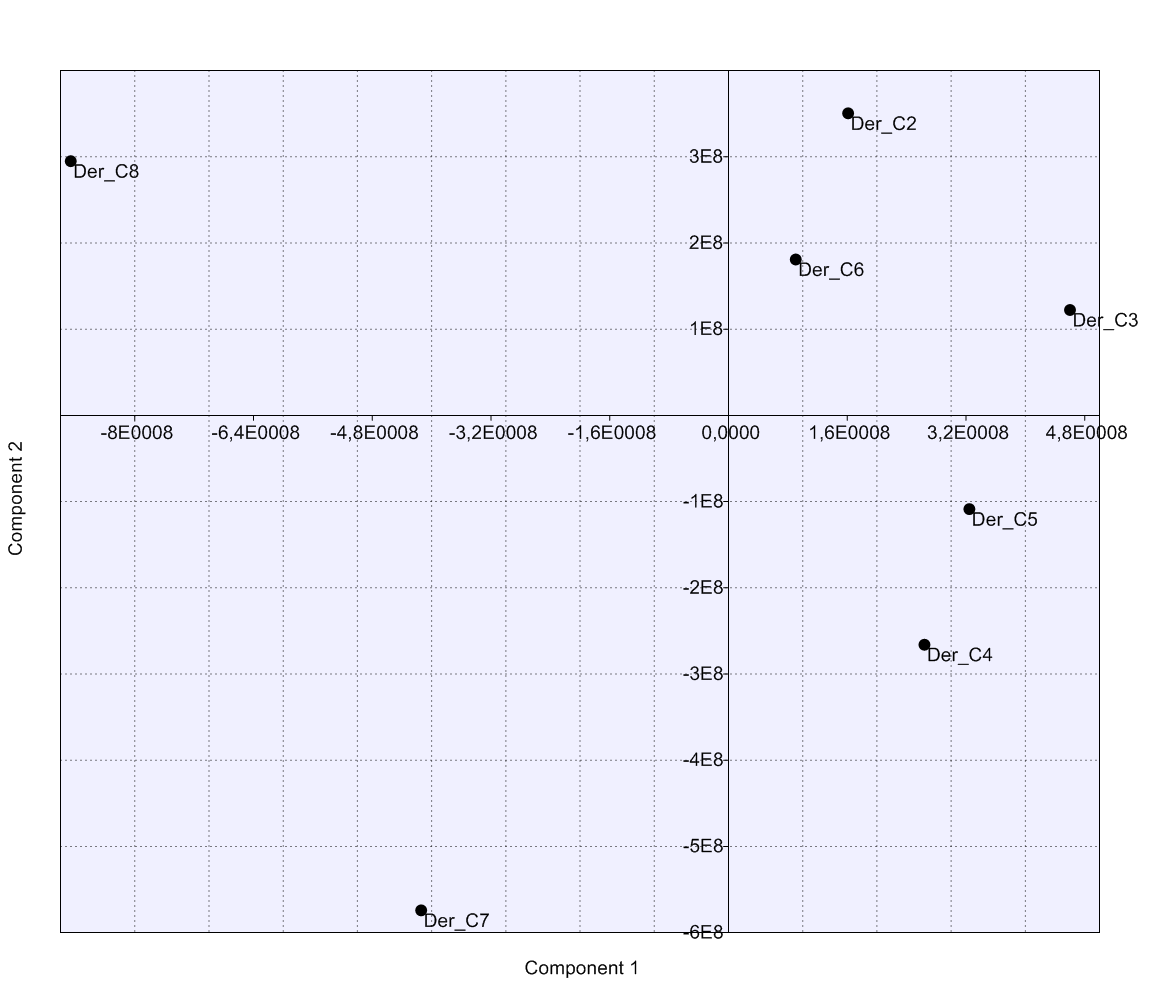


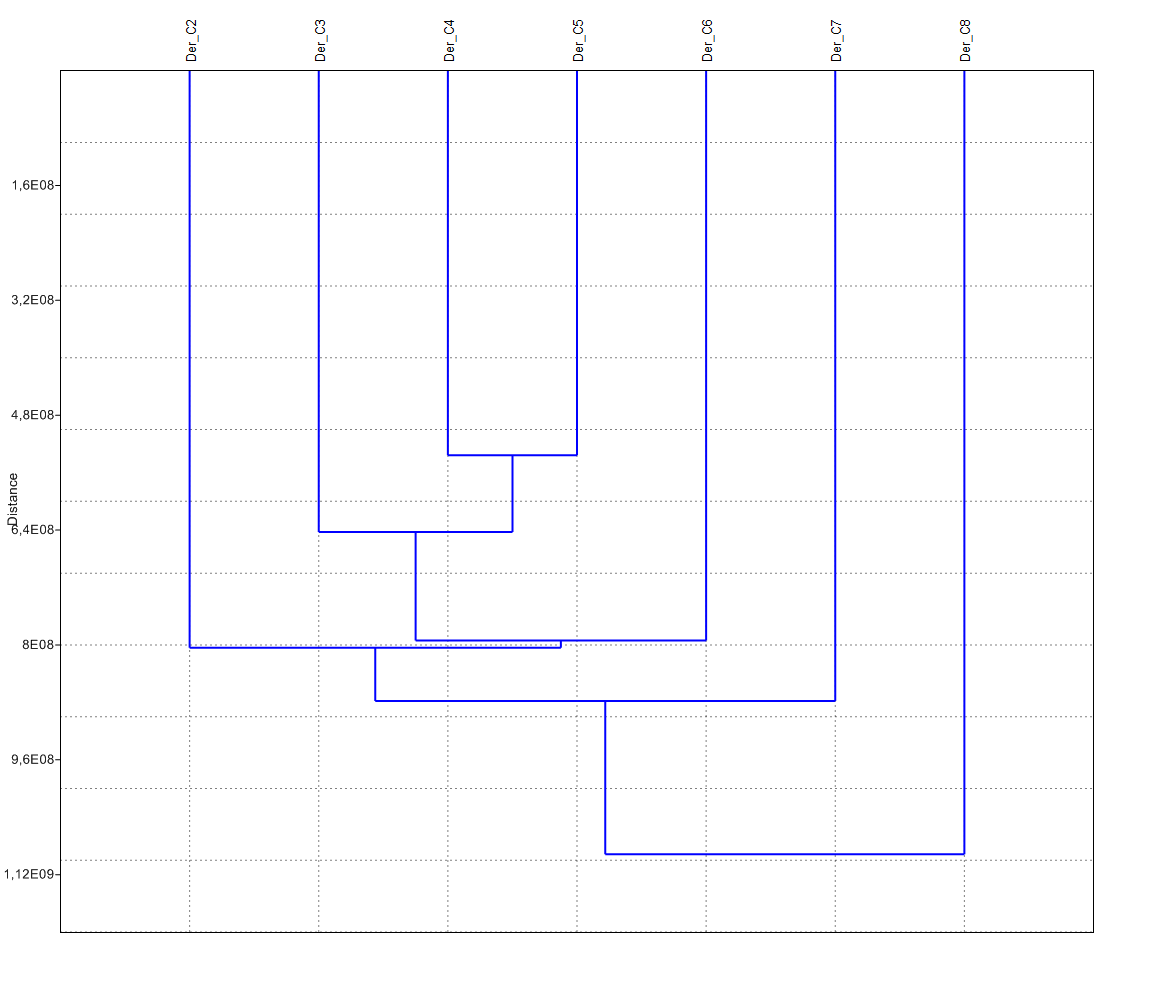


**I** *Platysternon megacephalum*


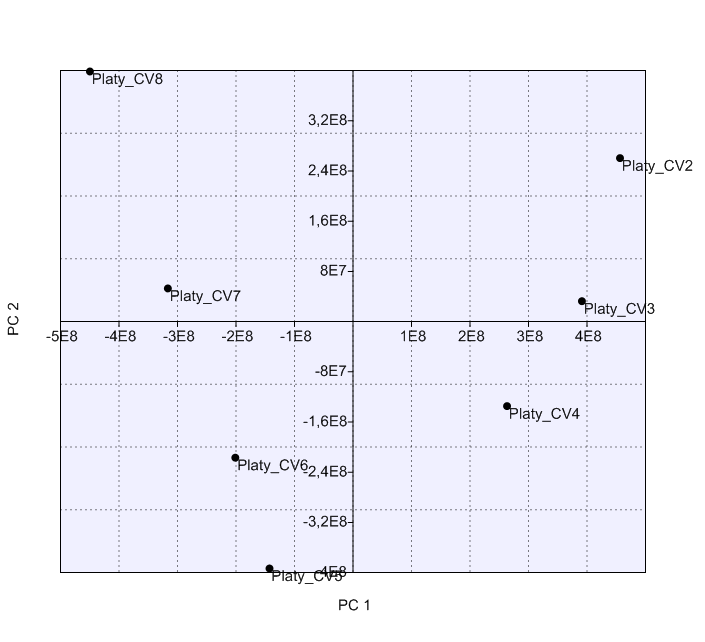


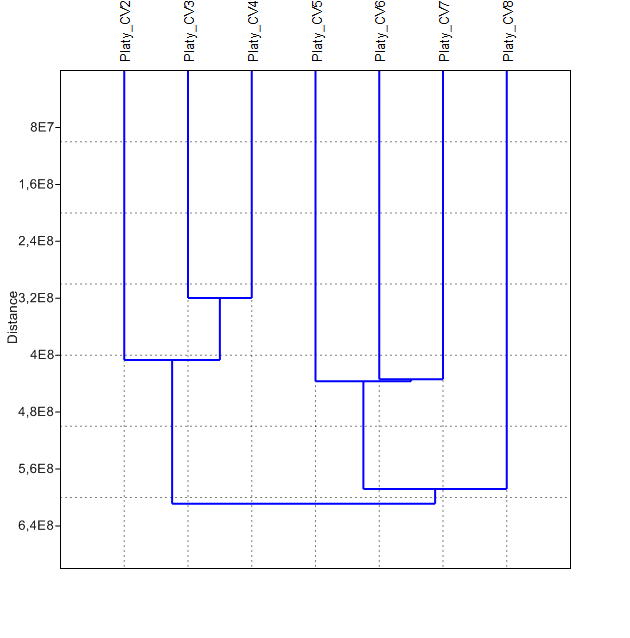


**J** *Testudo hermanni*


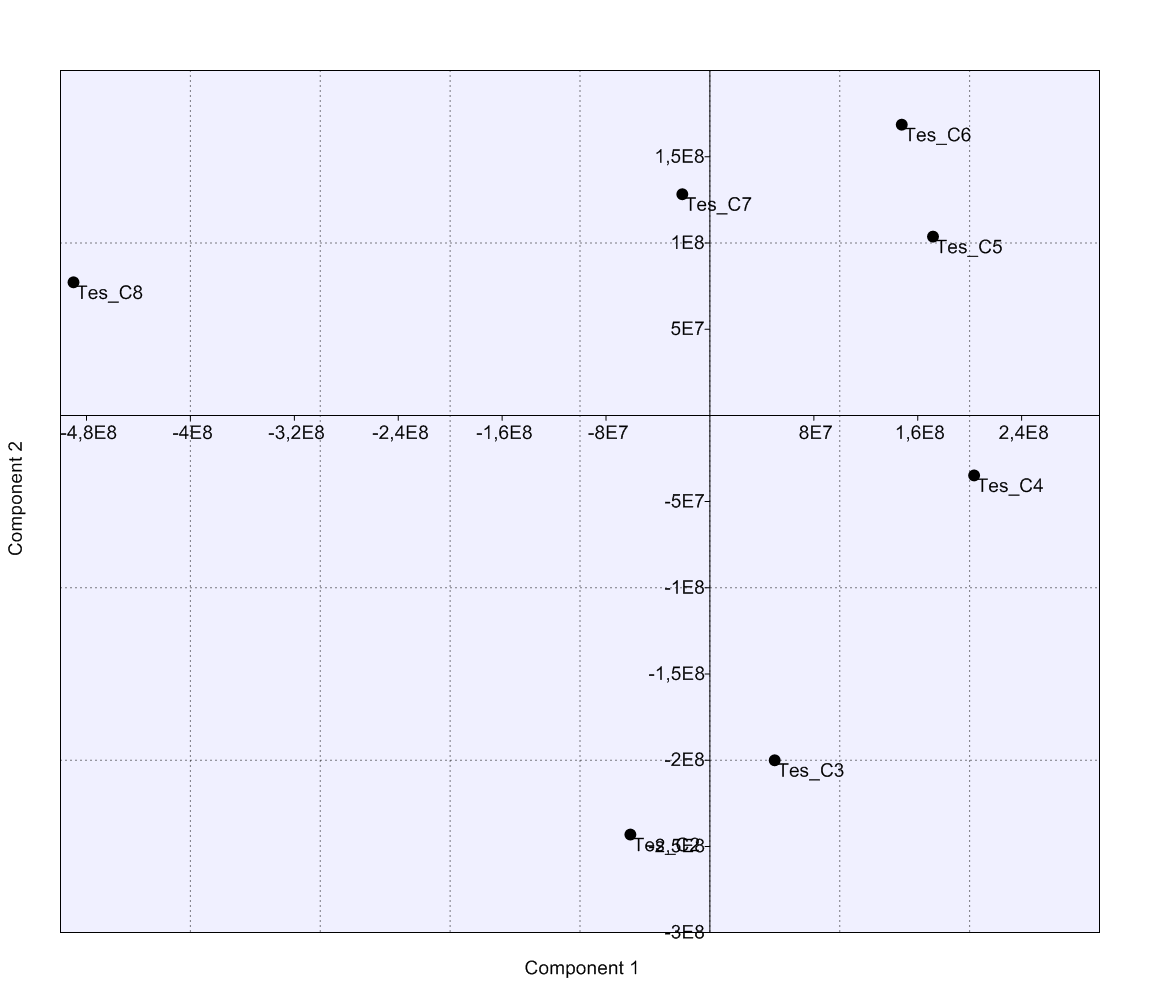


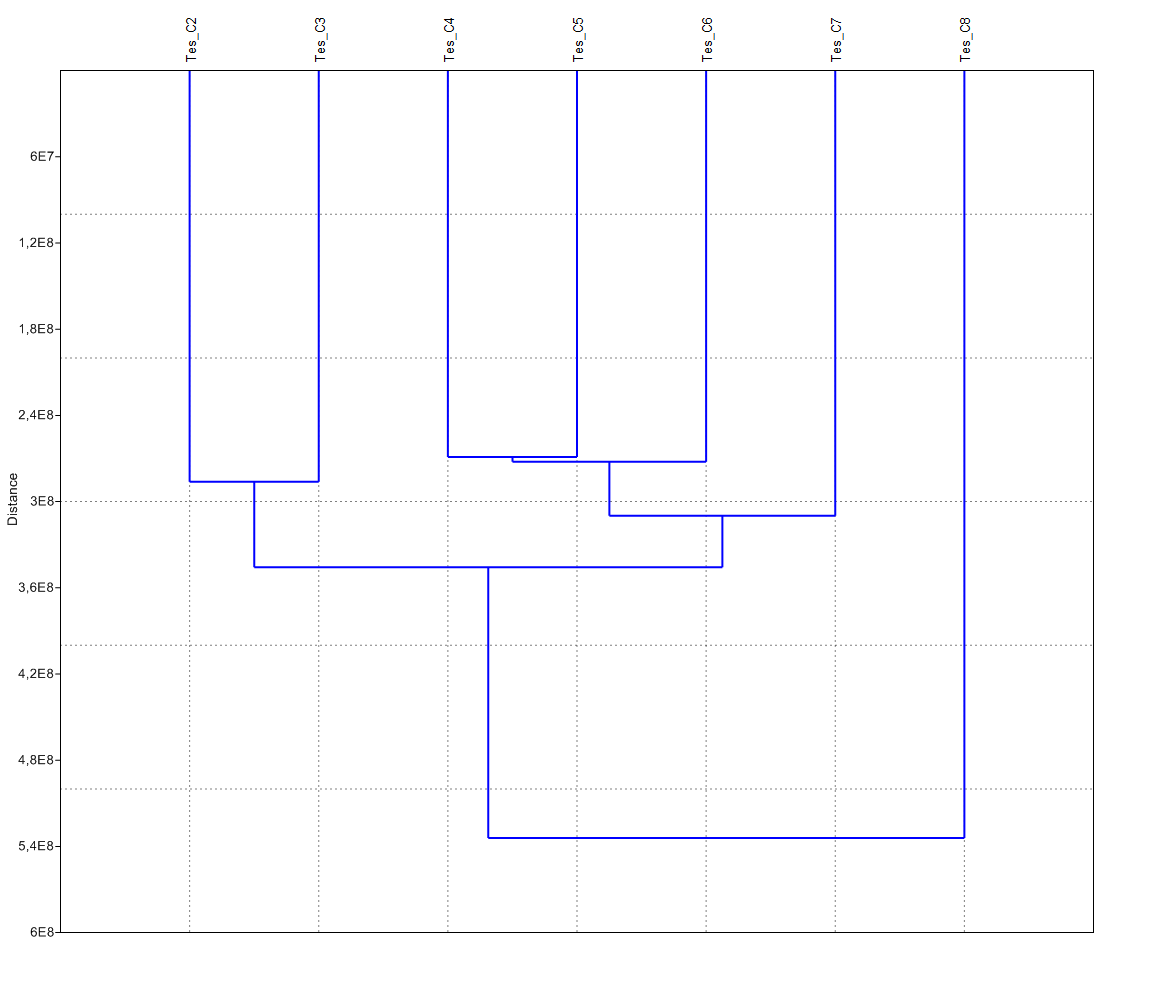


**K** *Malaclemys tomieri*


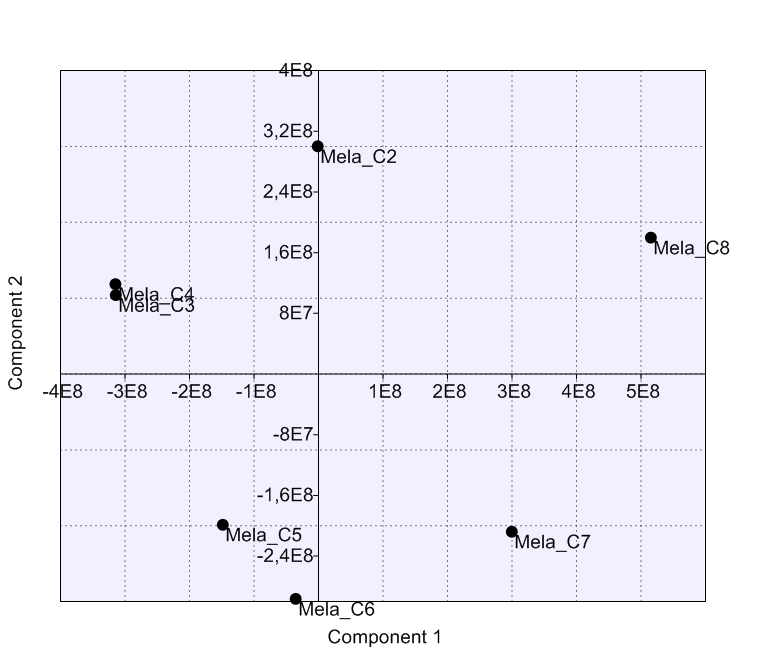


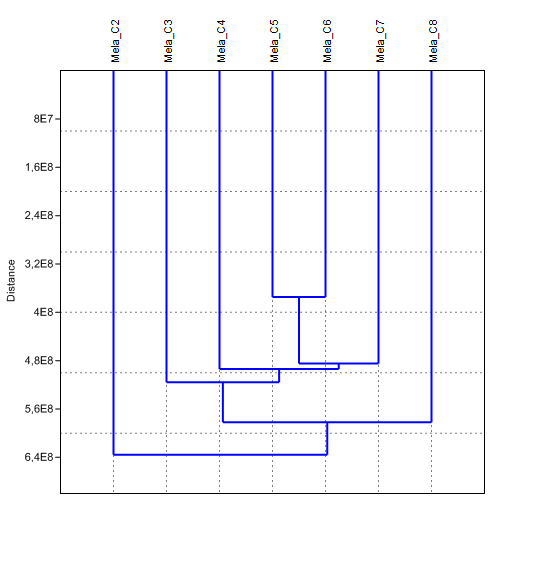


**Supplementary tables**

**Table S1**: Species analyzed in the present study. Collections: AMNHN = American Museum of Natural History, New York, USA; FNMH = Field Museum of Natural History, Chicago, USA; SMF = Senckenberg Forschungsinstitut und Naturmuseum Frankfurt, Frankfurt am Main, Germany.

| **taxonomic group** | **species** | **collection number** | **ID** |
| --- | --- | --- | --- |
| **stem turtles** | | | |
|  | ✝*Proganochelys quenstedti* | ANMH uncat (= cast of SMNS 16980) | IW921A |
|  | ✝*Meiolania platyceps* | AMNH 57984. Cast of Australian Museum (Late Pleistocene. Lord Howe Island. New South Wales. Australia. *Meiolania platyceps*) | IW924A |
|  | ✝*Naomichelys speciosa* | FNMH PR273 | IW931 |
| **Pleurodira** | | | |
|  | *Hydromedusa tectifera* | SMF 70500 | IW1040 |
|  | *Phrynops geoffroanus* | SMF45470 | IW935 |
| **Cryptodira** | | | |
|  | *Macrochelys temminckii* | Teaching collection of Fachbereich Geowissenschaften Tübingen | IW1113 |
|  | *Kinosternon scorpioides* | SMF71893 | IW936 |
|  | *Dermatemys mawii GRAY* | SMF59463 | IW1035 |
|  | *Platysternon megacephalum* | SMF69484 | IW920 |
|  | *Testudo hermanni* | SMF71882 | IW1045 |
|  | *Malaclemys ("centrata“) terrapin* | SMF36419 | IW934 |

**Table S2**: Definition of landmarks (LM) applied in the present study. Compare to figure 2.

| **LM** | **position** | **description** |
| --- | --- | --- |
| 1 | Median | Embayment between the anterior zygapophyses |
| 2 | Right | Distal most projection of the anterior zygapophysis |
| 3 | Left |
| 4 | Right | Embayment between the distal most projection of the anterior zygapophysis and the anterior central articular process |
| 5 | Left |  |
| 6 | Right | Posterolateral most tip of the articulation surface of the anterior central articular process |
| 7 | Right | Distal projection or embayment in a double joint of the anterior central articular process; between 4 and 8 in simple joints |
| 8 | Median | Median projection or embayment in the anterior central articular process |
| 9 | Left | See 7 |
| 10 | Left | See 6 |
| 11 | Left | Distal most projection of the posterior zygapophysis |
| 12 | Median | Embayment between the posterior zygapophyses |
| 13 | Right | See 11 |
| 14 | Left | Embayment between the posterior zygapophysis and the posterior central articular process |
| 15 | Right |
| 16 | Left | Anterolateral most tip of the articulation surface of the posterior central articular process (compare to 6) |
| 17 | Left | Distal projection or embayment in a double joint of the posterior central articular process; between 16 and 17 in simple joints (compare to 7) |
| 18 | Median | Median projection or embayment in the posterior central articular process; together with 19 and 21 in simple joints (compare to 8) |
| 19 | Right | See 17 |
| 20 | Right | See 16 |
| 21 | Median | Distal most projection of the hypophyseal crest |
| 22 | Left | Distal most tip of the transverse process |
| 23 | Right | See 22 |
| 24 | Median | Meeting point of the dorsal crests of the anterior zygapophyses |
| 25 | Median | Meeting point of the dorsal crests of the posterior zygapophyses |
